# Long-Term Variability in Visual Processing versus Perceptual Stability

**DOI:** 10.1523/ENEURO.0344-25.2026

**Published:** 2026-06-19

**Authors:** Laura Bock Paulsen, Laura Masaracchia, Francesca Fardo, Christine Ahrends, Diego Vidaurre

**Affiliations:** ^1^ Department of Linguistics, Cognitive Science and Semiotics, Aarhus University, Aarhus C 8000, Denmark; ^2^ Center of Functionally Integrative Neuroscience, Department of Clinical Medicine, Aarhus University, Aarhus C 8000, Denmark; ^3^ Oxford Centre for Integrative Neuroimaging (OxCIN), University of Oxford, Oxford OX3 0BP, United Kingdom; ^4^ Oxford Centre for Human Brain Activity (OHBA), Department of Psychiatry, University of Oxford, Oxford OX3 0BP, United Kingdom

**Keywords:** decoding, magnetoencephalography, variability, visual processing

## Abstract

Our brain is in constant change due to neural plasticity, but, still, our experience of the world feels relatively stable to us. Focusing on visual processing, we hypothesize that brain responses to stimuli may change over long periods of time, but in a way that is orthogonal to the dimensions that are relevant to stimulus category discrimination. To test this hypothesis, we acquired and analyzed a magnetoencephalography (MEG) dataset containing recordings from one female adult participant, with several scanning days spanning over 6 months. The participant passively attended to visual stimuli with the same stimulus presented within each session. We demonstrate that the specific scanning day can be predicted from the brain responses in a simple passive viewing paradigm, suggesting continuous temporal changes in neural activity over long time scales. However, information from one scanning day could be used to robustly decode the animacy of objects on a different scanning day, and importantly, decoding accuracy did not suffer with increasing time intervals between scans. That is, cross-decoding accuracy remained stable over months, despite between-scanning-day variability. These findings suggest that while processing of visual stimuli shows variability over long time scales, the core neural structure underlying object recognition remains stable and non-stimulus-specific. The results were validated in the open-access THINGS-MEG dataset, which employs a similar paradigm but covers a shorter longitudinal timespan. We find similar results across the four additional participants.

## Significance Statement

While prior research has shown that neural responses to the same stimuli can change over short timescales (seconds to days), it remains unknown whether such changes impact higher-level representations, such as object categories, over longer periods. Using longitudinal magnetoencephalography data collected from a single individual across 6 months, we show that responses to repeated visual stimuli drift over time, yet category-level representations remain robust. These findings demonstrate that the brain maintains stable perceptual representations despite gradually changing sensory responses, with implications for theories of neural variability, long-term decoding, and individual-level brain tracking.

## Introduction

Without our awareness, our brain’s state changes from moment to moment. This variability occurs at multiple levels: from single cells (Abbott and Nelson, 2000; Benda, 2021) to networks of neurons (Fontanini and Katz, 2008; Turrigiano, 2011; Marder, 2012; Goris et al., 2014), leading to differences in the brain’s response to even simple, repeating stimuli (Dinstein et al., 2015; Stauch et al., 2021). However, we consistently and reliably recognize and categorize objects (Biederman, 1987), a testament to the brain’s ability to maintain perceptual stability despite neural variability (DiCarlo et al., 2012).

This temporal (trial-by-trial) neural variability raises important questions about how stable mental representations can be formed and maintained. Yet, much of our understanding of object categorization comes from studies that capture participants’ responses in a single session, which restricts the investigation of variations in visual processing over time scales longer than the duration of the experiment. These studies have provided valuable insights into how mental representations of objects evolve from stimulus onset to the first few hundred milliseconds that follow. To characterize these dynamics, they often use machine learning to decode object categories from neuroimaging data (Carlson et al., 2013; Cichy et al., 2014; Isik et al., 2014; Brandman and Peelen, 2017; Contini et al., 2017; Grootswagers et al., 2017), exploring when these categories are processed in the brain by seeing how trained classifiers generalize across time points within trials and conditions. However, these designs do not afford investigation of whether and how such representations evolve over days, weeks, or months.

This question is especially timely in light of growing evidence that neural variability does not merely reflect random noise but may arise from drifts in neural representations. Much of this evidence was found in mice, where progressive drift was found in tuning and activity of cells to natural stimuli over time ranging from minutes to weeks (Deitch et al., 2021; Marks and Goard, 2021; Ebrahimi et al., 2022).

In humans, a recent fMRI study demonstrated representational drift in the primary visual cortex (V1) by training encoding models to characterize voxel-level tuning to visual stimuli and testing their generalization across sessions over the span of a year (Roth and Merriam, 2023). The models’ predictive performance declined as the interval between training and testing sessions increased, suggesting that the underlying neural representations slowly drifted over time. Notably, however, the representational similarity structure among individual scene stimuli remained stable across sessions, suggesting preserved stimulus-space geometry despite drift in activation patterns.

What remains unclear is whether such drift extends beyond stimulus-specific geometry to impact higher-level representational structure, such as object category representations that generalize across exemplars, or whether these more abstract representations remain stable despite lower-level fluctuations. Furthermore, it is unknown how neural responses measured at higher temporal resolution evolve over time scales of weeks or months.

Following the spirit of the MyConnectome dataset in fMRI (Poldrack et al., 2015) and using a personalized headcast to improve signal-to-noise ratio, we recorded magnetoencephalography (MEG) data from one participant while they viewed images of animate and inanimate objects, with multiple experimental repetitions spread over 6 months. Using machine learning methods, we investigated whether the processing of identical visual stimuli changes over time and how this influences object categorization. We show that the evoked MEG response to visual stimuli does evolve over long time scales (i.e., days to months), but these changes do not impact cross-scanning day decoding accuracies. These results indicate that these changes occur in dimensions unrelated to the neural patterns that drive object categorization; that is, they are not stimulus-specific, therefore allowing us to perceive the world coherently despite the neural variability.

## Materials and Methods

### Stimuli and procedure

**Figure 1.**
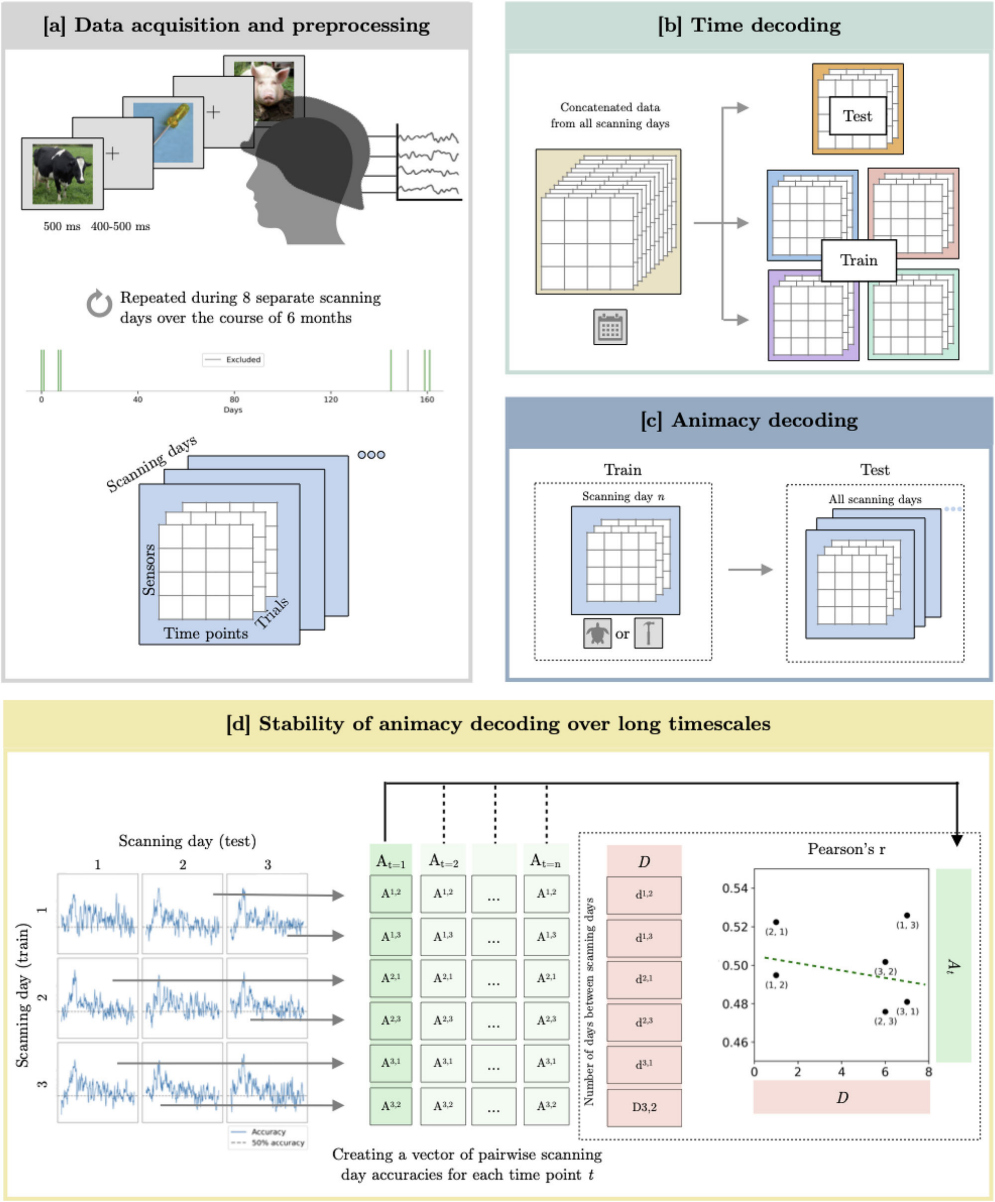
Data acquisition and analysis. ***A***, MEG recordings were obtained from a single participant presented with visual stimuli. The participant received identical visual stimuli whilst in the scanner during eight separate scanning days spanning 6 months. ***B***, To investigate if processing of identical stimuli varies over long time scales, we predicted both the specific scanning day and its position in the sequence of scanning days, indicating the repetition of the experiment for the participant. ***C***, We investigated if the long-term variations in the data are stimulus-dependent, by decoding whether the stimuli were animate (e.g., a dog) or inanimate (e.g., a table) both within and across scanning days. ***D***, To assess animacy decoding stability over longer time scales, we extracted the accuracy for each time point within the trial from stimuli onset to 1,000 ms after for each pair of scanning days (*A*_*t*_). The stability of decoding is explored by constructing a vector with the number of days between each scanning day and calculating Pearson correlations between the number of days and accuracies for each time point.

We acquired a dataset containing recordings from one single female adult subject. The dataset comprises recordings of neural responses using MEG from a total of eight scanning days, each with multiple sessions, over the course of 6 months. During each session within a scanning day, the participant viewed 118 different real-world images. Each image was presented 4 times each session lasting ∼13min. Multiple sessions were completed each scanning day with a small break between (Fig. 1*A* and Table 1). This resulted in more than 10,000 image presentations. One of the scanning days was cut short and was excluded from further analysis as a result of having too few trials. The participant was instructed to merely attend to the stimuli presented, consisting of 118 images (Cichy et al., 2016) with 27 images of animals (e.g., dog, bird, frog, and polar bear) and 91 inanimate objects (e.g., washing machine, strawberry, car, and table). Each image was presented for 500 ms, followed by a 400–500 ms fixation cross (Fig. 1*A*). For all analyses, all animate images and an equal number of inanimate stimuli were included.

**Table 1.** Overview of scanning days and number of presentations per stimulus (118 images total)

Scanning day	Days after first scan	Presentations per stimulus	Total recording duration
1	0	12	40.95 min
2	1	12	39.43 min
2	7	16	51.56 min
3	8	14	45.65 min
4	145	12	39.23 min
5*	152	6	19.5 min
6	159	14	45.24 min
7	161	18	57.97 min

Scanning day 5 (*) was excluded from further analysis as the scan was ended early, resulting in a substantially lower number of image presentations.

### Acquisition and preprocessing of MEG data

MEG recordings were obtained using an Elekta Neuromag MEG system (Elektra Neuro-mag Oy) inside a magnetically shielded room, equipped with 102 magnetometers and 204 gradiometers. The data was acquired using a headcast 3D printed to fit the shape of the participant’s head from a rendering of structural MRI data (Meyer et al., 2017). This ensured consistent head positioning across scanning days and sessions within a scanning day relative to the sensors and minimal movement during data collection. The position of the MEG chair within the MSR and the height was marked such that it could be placed in the same position before each session. Between sessions within a scanning day, the participant exited the MEG dewar and headcast during short breaks and was repositioned in the headcast before the next session. Signals were sampled online at 1,000 Hz.

The preprocessing of MEG data was carried out in MNE-python (Gramfort et al., 2014) and consisted of the following ordered steps. Signal space projection was applied using projectors estimated from empty-room recordings to remove environmental noise components. Bad channels were identified by visual inspection and interpolated to ensure an equal number of features across sessions (between 1 and 11 removed, *μ* = 6.06). The data were high-pass filtered at 1 Hz to reduce slow drifts and low-pass filtered at 40 Hz using linear-phase finite impulse response filters implemented in MNE-Python. Independent component analysis (ICA) was conducted to identify and remove artifacts. Cardiac and ocular components were manually identified and removed, based on inspection of component topographies and time courses (1–4 components per session; *μ* = 2.36). The data were then segmented into epochs from stimulus onset to 1 s post-stimulus and resampled to 250 Hz.

The preprocessing was done separately for each session within a scanning day, however all subsequent analyses concatenate sessions within each scanning day.

### Analysis

To investigate if and how the processing of identical visual stimuli changes over long time scales, and whether these changes are related to the patterns that allow for stimulus discrimination, we conducted two analyses.

First, we attempted to decode the scanning day and scanning sequence order from the MEG brain responses (Fig. 1*B*). The idea is that if there is a systematic, monotonic change in the signal throughout time (in the order of weeks or months), then the signal should be statistically significant predictor of time itself—either scanning order or day. On the contrary, if there is not any long-term variability in the signal (or if the change is not monotonic), then we should not be able to predict time above chance.

Second, we trained models to discriminate between animate and inanimate objects (e.g., a pencil) using data from each scanning day separately. These models were tested both within the same scanning day and across different scanning days (i.e., training on one scanning day and testing on another) to evaluate how accuracy is affected by the temporal distance between scanning days (Fig. 1*C*,*D*). If we found that accuracy declined as the training and testing sessions become farther apart, it would indicate that the structure of visual processing slowly changes over time.

#### Decoding scanning day and scanning order

To evaluate whether the processing of identical stimuli varies over long time scales, we used linear ridge regression (McDonald, 2009) as implemented in scikit-learn (Pedregosa et al., 2011) to predict two variables from the MEG data: the calendar day of the scan and its position within the sequence of scanning days (scanning order). These two variables are not perfectly correlated, as the intervals between scanning days varied. Thus, calendar day captures absolute time, while scanning order reflects the repetition of the experimental paradigm.

Decoding was performed separately at each time point from stimulus onset to 1,000 ms post-stimulus. At a given time point, the feature vector for each trial consisted of the MEG signal across all gradiometers and magnetometers. Thus, each time point yielded an independent regression model, allowing us to track when information about the scanning day and scanning order is present in the evoked response.

To evaluate whether these variables could be significantly decoded from the MEG data, we employed a bootstrapping framework. A total of 2,000 bootstrap samples were generated, each containing 200 randomly selected animate trials per scanning day. For each bootstrap sample, a ridge regression model was trained and evaluated using fivefold cross-validation (CV) with 280 trials per fold, at each time point from stimulus onset to 1,000 ms post-stimulus. The folds were constructed to ensure an equal number of trials from each scanning day.

The model’s performance was quantified using the explained variance, computed at each time point as:

$$\text{EV}=1-\frac{\sum (y-y_{\text{\textbackslash{},pred}}{)}^{2}}{\sum (y-\bar{y}{)}^{2}},$$

where *y* represents the ground-truth scanning day or scanning order, *y*_pred_ is the out-of-sample predicted scanning day or scanning order and $\bar{y}$ is the mean of *y*.

Given our bootstrap samples, we performed a bootstrap-based hypothesis test. The null hypothesis amounts to no relationship between MEG data and the target variables.

For each time point, we computed bootstrap *p*-values by determining the proportion of bootstrap samples in which the explained variance is above 0. False discovery rate (FDR) correction was applied using the Benjamin–Hochberg method to control for multiple comparisons (Benjamini and Hochberg, 1995).

#### Cross-scanning day decoding of stimuli animacy

To investigate whether the changes observed in the brain responses over longer time scales (as assessed by the methods described in the previous subsection) are stimulus-specific, we utilized a cross-scanning-day decoding approach. For each scanning day and time point within the trial, decoders were trained to discriminate animate and inanimate stimuli, a contrast that has previously been shown to exhibit good decodability (Carlson et al., 2013; Cichy et al., 2014; Brandman and Peelen, 2017; Grootswagers et al., 2017). Decoding analysis was performed using linear discriminant analysis (Zhao et al., 2024) using scikit-learn (Pedregosa et al., 2011) and repeated for each time point within the trial. At a given latency, each trial was represented by the MEG signal across all sensors at that time point. Tenfold CV was implemented with ∼60 trials per fold. In each fold, decoders were trained on data from a subset of trials from one scanning day and tested on data from all scanning days across all time points, resulting in a 7 scanning days ×7 testing days ×250 time points matrix after averaging over CV folds.

The data from the training folds were standardized for each feature separately, and used to train the decoder. The test fold was standardized using the mean and standard deviation of the training set, before making predictions using the decoder.

To assess whether discrimination of object categories remains consistent across time, we looked at cross-scanning-day accuracies. We constructed a vector indicating the number of days between the data used for training and the data used for testing. We then calculated the Pearson correlation between this vector and the accuracies, with the goal of seeing whether cross-scanning day decoding accuracies decrease when the sessions are farther apart in time. This was repeated for every time point (Fig. 3). We conducted cluster-based permutation testing to compare the observed correlation with a distribution of 10,000 correlations computed after permuting the vector representing the number of days between train and test data, allowing us to establish whether the observed relationship between temporal distance and accuracy is statistically significant. If the correlation in the original data significantly differs from the permuted, it would suggest that the longitudinal changes we observe are stimulus-specific.

#### Additional dataset

To validate and compare our results, we repeated the analyses on a similar dataset from Hebart et al. (2023). The THINGS-MEG dataset consists of MEG recordings from four participants who viewed images drawn from the THINGS database (Hebart et al., 2019), which comprises 1,854 object concepts represented by 26,107 naturalistic images. The data were recorded using a CTF 275 MEG system (CTF Systems) with a whole-head array of 275 radial first order gradiometer channels.

Each participant was shown a representative subset of these images, distributed across 12 separate recording sessions. Each image was presented for 500 ms followed by a 1 ± 0.2 s fixation cross. Similar to our dataset, participants wore custom headcasts, and head positions were found to be consistent both within and across scanning days.

The THINGS-MEG dataset differs from our dataset in two key respects. First, in our dataset, the same images were presented multiple times within each scanning day, whereas in THINGS-MEG each image was shown only once. Second, our dataset spans a longer total recording period (161 d), whereas THINGS-MEG was collected over shorter intervals of 29, 32, 43, and 72 d for participants 1–4, respectively.

*Preprocessing:* For the THINGS-MEG dataset, we did not apply ICA. Four channels that were identified as bad by the original authors were removed, leaving 271 channels for subsequent analyses (Hebart et al., 2023).

To account for differences between the number of trials on each scanning day, a few modifications are made to the analyses.

*Decoding scanning day and scanning order:* Each bootstrap contains 150 rather than 200 images from each scanning day, as 203 animate images were presented each scanning day (compared to the minimum of 294 in our collected data). As the dataset contains 12 scanning days rather than 7, this results in 360 trials per CV fold.

### Code availability

All analysis code is available as Extended Data and on Github (https://github.com/laurabpaulsen/VisualVariability).

10.1523/ENEURO.0344-25.2026.d1 Data 1 Download Data 1, ZIP file.

## Results

### Variability in visual processing over longer time scales

Our first question is whether there is a systematic change over long periods of time in the patterns of activity evoked by visual stimuli. For this, we trained machine learning models to predict, using the MEG brain responses, the order of the scans (or, equivalently, the specific day when these happened).

**Figure 2.**
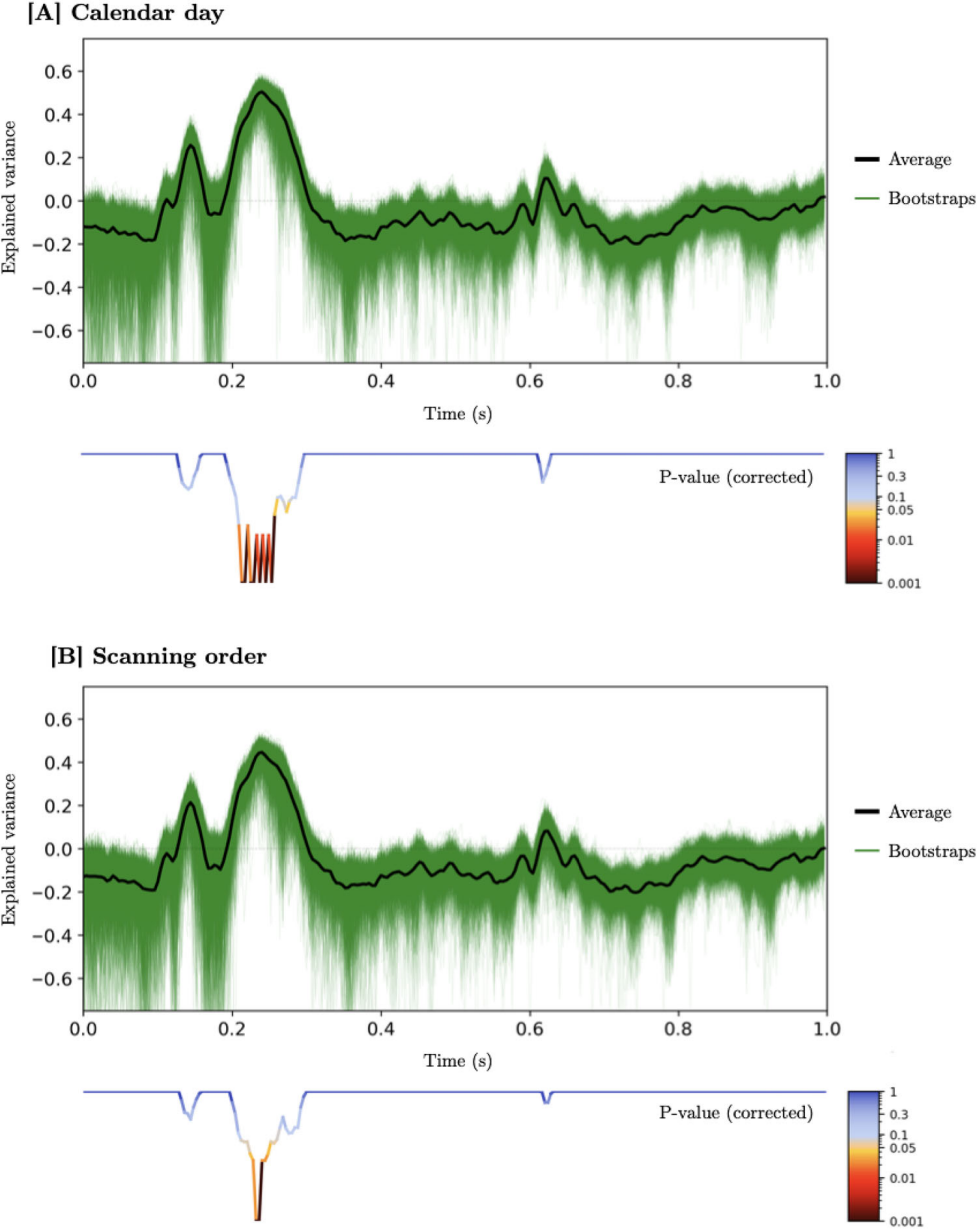
Decoding calendar day and scanning order. Explained variance of model trained on bootstrap samples to predict calendar day of the scan (***A***) and scanning order (***B***). Thin green lines represent the explained variance for each individual bootstrap sample. The black line shows the average across bootstrap samples. The explained variance can take negative values when the model performs worse than the baseline model (which is here the mean). The colored line underneath shows the *p*-value (corrected for multiple comparisons using Benjamin–Hochberg method). See Extended Data Figures 2-1 and 2-2 for the same analyses using the THINGS-MEG dataset.

As shown in Figure 2, we see an increase in explained variance approximately 100 ms after the presentation of the stimuli, peaking at 240 ms for both calendar day and scanning order.

10.1523/ENEURO.0344-25.2026.f2-1 Figure 2-1 Decoding scanning day results for THINGS-MEG dataset. Download Figure 2-1, TIF file.

10.1523/ENEURO.0344-25.2026.f2-2 Figure 2-2 Decoding scanning day number results for THINGS-MEG dataset. Download Figure 2-2, TIF file.

**Figure 3.**
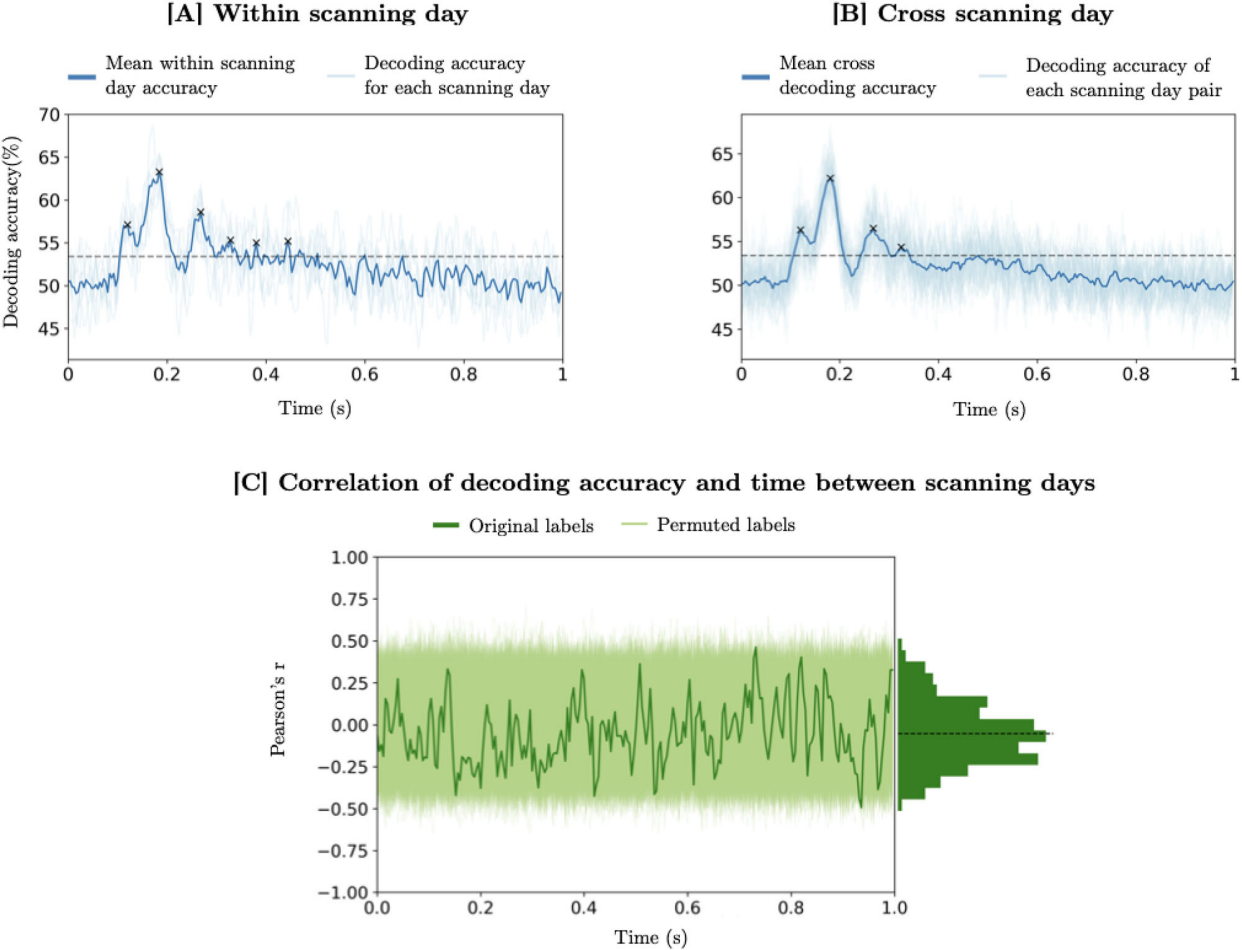
Decoding animacy within and across scanning days. Time courses of decoding accuracies for determining the animacy of stimuli, distinguishing between animate objects (e.g., dog) and inanimate objects (e.g., washing machine), both within (***A***) and across (***B***) scanning days. The dashed line indicates chance-level accuracy (given the number of trials and *α* = 0.05). The dark blue line shows the mean accuracy, whereas the light blue lines show the within-scanning day decoding accuracies for each scanning day (***A***) and the cross-decoding accuracy for each pair of scanning days (***B***), respectively. Note that the cross-scanning-day average appear smoother as it represents the average over a larger number of pairs compared to within-scanning-days. ***C***, Time-resolved analysis of long-term stability of animacy decoding. The *x*-axis shows post-stimulus time (0–1 s). At each time point, we computed the Pearson correlation across all pairs of scanning days between (i) cross-day decoding accuracy at that time point and (ii) the temporal distance (in days) separating the corresponding scanning days. Negative correlations would indicate that decoding performance decreases as the temporal distance between recordings increases. Light green lines show correlations obtained using permuted scanning-day distances (null distribution). No significant clusters (*p* < 0.001) were identified. See Extended Data Figure 3-1 for results using the THINGS-MEG dataset.

10.1523/ENEURO.0344-25.2026.f3-1 Figure 3-1 **Correlation of decoding accuracy and time between scanning days for THINGS data**. Same analysis as in Figure 3C. No significant clusters (*p* < 0.001) were identified for any of the THINGS-MEG participants. Download Figure 3-1, TIF file.

For both calendar day and scanning order, we found time points where the explained variance is significantly larger than 0. We find significant time points for participants 1, 2, and 4 of the THINGS dataset (Extended Data Figs. 2-1 and 2-2). This demonstrates that the evoked MEG responses to visual stimuli show some systematic longitudinal changes over long periods of time. Given the similar results for calendar day and scanning order, it is possible that these changes reflect a combination of cumulative experience across scanning days, such as increasing familiarity with the stimuli, and more general temporal dynamics not directly related to the task.

### Consistency in categorization of object category over longer time scales

Having established that brain responses exhibit a systematic change over time, we questioned the extent to which these changes are stimulus-dependent; or else, if they occur in orthogonal dimensions to the patterns that allowed us to decode between stimulus categories. If the former is true, we would observe, as a result of changes in stimulus processing, (1) differences between within and cross-scanning day decoding and (2) worse performance of decoders trained and tested on scanning days that are farther apart, compared to those that are closer together in time.

As a preliminary step, and consistent with findings from previous studies (Carlson et al., 2013; Cichy et al., 2014; Brandman and Peelen, 2017; Grootswagers et al., 2017), we verified that it is possible to decode the animacy of the stimuli within scanning day (Fig. 3*A*). Mean decoding accuracy peaked at 63.0% (sd = 1.89) 172 ms post-stimulus, and all scanning days exceeded the chance threshold of 53.4%, determined by the number of trials and a significance level of *α* = 0.05 (Combrisson and Jerbi, 2015).

We then assessed whether decoders (as descriptors of visual processing mechanisms) are able to generalize across scanning days; that is, when training on data from one day and testing on another day. Figure 3*B* shows that they can, with a peak average accuracy of 61.8% (sd = 2.68) 180 ms post-stimulus.

Last, we examined whether decoders degrade their performance in any way when the training session is further away from the testing session. For this, we assessed the stability of object categorization over long time scales (Fig. 3*C*) using a cluster-based permutation test. No significant clusters were found, indicating that increased time between training and testing was not associated with poorer performance. No effect was found in either of the four participants in the THINGS-MEG dataset either (Extended Data Fig. 3-1).

Overall, these findings suggest that, at least within the time frame examined, the neural representations of object categories are largely stable, and that, while there are longitudinal changes, these are not stimulus-specific.

## Discussion

While much is known about visual object recognition, we know less about its stability across longer time scales. Here, we analyze neural responses to visual stimuli using decoding analyses. First, we show that both the calendar day and the order of the scans can be predicted from the brain responses, emphasizing that the response to identical visual stimuli changes over time in a way that is detectable using MEG. Extending these findings, we analyzed an additional four participants in the THINGS dataset. Notably, no exact stimulus images were repeated (although pictures of the same object categories were shown). Despite the absence of image repetition both scanning day and scan order could still be predicted from the MEG responses in three of the four participants. This indicates that the temporal structure in the neural data is not driven by stimulus-specific repetition but reflects broader changes that generalize across stimuli.

Second, we show that the animacy of an object can be decoded from MEG signals, not only within a single experimental session but training and testing on data from different scanning days. Cross-scanning day decoding remained stable over time and did not vary systematically with the temporal distance between scanning days. This stability was also observed in the four THINGS participants.

Taken together, these findings suggest that, while there are variations across time, the basic structure of object recognition remains consistent, enabling cross-scanning day decoding irrespective of temporal gaps.

Crucially, while prior studies have demonstrated short-term fluctuations or plasticity in neural responses to repeated visual stimuli over minutes to days (Stauch et al., 2021; Miyamoto et al., 2025), our results show that category-level representations remain robust over months. This highlights the stability of higher-order perceptual representations in the face of ongoing changes in the underlying sensory responses.

An important open question is whether we can use basic perceptual tasks in M/EEG as “neural fingerprint,” akin to fMRI (Finn et al., 2015). For this, two requirements need to be met. First, the signal between participants needs to be distinct enough, which has been shown previously on single-session MEG data (Colenbier et al., 2023). Second, that the information of interest needs to be stable enough over time (Gratton et al., 2018). Here, we show that the latter was largely the case for passive vision by using a new data set where a single subject performed the task repeatedly over the course of months.

Using MEG as a fingerprint has several potential benefits. It could enable the tracking of individual neural stability over time in a controlled manner, shedding light on changes due to learning, development, or pathology. MEG’s high temporal resolution might also reveal dynamic individual differences not captured by fMRI. These findings highlight the promise of MEG for individualized and long-term brain mapping.

This study has limitations that will be addressed by future work. A key feature of this dataset is that it contains many recordings from the same participant, which allows for detailed analysis of temporal stability within an individual. However, the generalizability of the results should be confirmed across multiple participants so that we can positively determine whether the observed decoding stability and temporal effects are robust at the group level. This would also allow us to explore potential inter-individual differences in these patterns. Another limitation pertains to the analysis of how visual processing differs over long time scales. The observed systematic longitudinal changes in the evoked MEG response may not solely reflect differences in brain activity but could also be influenced by external factors specific to the scanning days. For instance, if an unrelated source of noise (e.g., from a nearby construction site) was present during the first half of the scanning days but not the last, this could enhance model predictions by leveraging this artifactual effect. While we verified that this was not the case to the extent of our powers, this cannot be categorically ruled out. Notably, in both the original participant and two THINGS participants (1 and 4), the effects were limited to a narrow post-stimulus window(∼200–300 ms), rather than being distributed uniformly across the trial, which is inconsistent with typical environmental noise sources.

In summary, our results show that while the brain’s response to identical visual stimuli changes over time, these changes occur in a manner that preserves the stability of key neural patterns, such as those related to object categorization. Overall, these findings provide a step toward understanding how the brain maintains stable yet flexible representations over time and highlight the potential of MEG as a tool for longitudinal brain mapping and individual neural fingerprinting.

## Synthesis

Reviewing Editor: Nicholas J. Priebe, The University of Texas at Austin Decisions are customarily a result of the Reviewing Editor and the peer reviewers coming together and discussing their recommendations until a consensus is reached. When revisions are invited, a fact-based synthesis statement explaining their decision and outlining what is needed to prepare a revision will be listed below. The following reviewer(s) agreed to reveal their identity: Andreas Ioannides. Note: If this manuscript was transferred from JNeurosci and a decision was made to accept the manuscript without peer review, a synthesis may not be available.

Two reviewers evaluated the manuscript "Long-term variability in visual processing versus perceptual stability." Both reviewers recognized that this manuscript provides and interesting and important perspective on the stability of visual representations, albeit from a single participant. The reviewers raised a number of questions related to the specific methods used in the experiment, the data analyses and the treatment of the prior literature. These issues are detailed below, which should be useful in revising your manuscript, if you decide to resubmit it to eNeuro or to another journal.

Major Issues:

1) Clarifications of methods employed

Stimuli and procedures

This subsection does not provide enough detail for what was done and when. There are many other ways to provide the information that the reader needs to have to appreciate what was done and how. The manuscript should more details and specific examples of the stimuli presented. We suggest the inclusion of a figure and a table. The figure should show the sequence of events and details during one recording day (preparation, time of resting state data before and after (if such data were recorded), and the chronology of the 8 days of measurement (including the one with insufficient trials). The Table should provide the duration of the scan, duration, number of presentations of each type and all details (is it 118 once so about 2 minutes in total? Different each day of recording, any ISI between images) any baseline recordings (e.g. resting state, eyes open/closed?).

Acquisition and preprocessing of MEG data

More details are needed for the use of headcast:

Lines 94-97: "The data was acquired using a headcast 3D printed to fit the shape of the participant's head from a rendering of structural MRI data [23]. This ensured similar positioning of the head in the scanner across scanning days and minimal movement during data collection."

The need to keep the head fixed is obvious for types of analysis which assume that the head is in precisely the same location on each day of recording. This will be the case source space analysis where source localization depends on knowing where the sensors are relative to the brain; it is also necessary if the identity of each sensor is preserved in the analysis across days, which I believe is the case in this work. To go further one should ask: what are the main reasons for using the headcast:

Is it to fix the location so it is known relative to the sensors? If yes, why not place localization coils on the cast - that would produce an excellent coregistration result. Which can also be used for co-registering to the brain.

Is it to restrict movement? If yes, this will only be true in the two horizontal conditions. However, if the vertical (sitting) position was used it will depend critically on how long the measurements lasted. It is also relevant if supine position was used, but not as critical.

The next line is a tautology, unless one of two explanations

Lines 99-100: "Signal space projections were projected out. ..."

a) Some projection was made. In which case the sentence should be "Signal space projections was used to project out .... ."

With the "..." stating what noise or other components were removed.

b) Signal space projection was used to transform different head locations to one for use across all runs/days, in which case more details should be explained.

Lines 100-101: "Bad channels were identified by visual inspection and interpolated to ensure an equal number of features across scanning days."

Please provide the range of how many bad channels were encountered across the different days of recordings.

Lines 102-106: "To better facilitate identification of artefacts [25], a stronger high-pass filter at 1 Hz was applied to the data. ... Additionally, the data was low-pass filtered at 40 Hz. ..."

What is meant by a "stronger high-pass filter at 1 Hz"? Reference 25 does not provide any information because it cannot be found (the title is missing from the definition provided).

The filtering confines the search to relatively low frequencies, so the results demonstrated are relevant to that range (1-40 Hz) band, some more information about the kind of filters used would help check whether the filtering has other consequences, for example if by strong filtering it is meant sharp cut-off.

Reaching the end of subsection 2.2, it is confusing for what pre-processing operations (filtering, eye and heart artifact eliminations) were visual inspection, signal space projection and PCA used. Could this be all put in one place and clearly defined what method was used for what pre-processing step?

Analysis

There is important information missing in this section. For the one equation provided there is no statement about which data were used. In section 2.2 it is stated that the recordings were made with the NeuroMa-Oy system with 305 channels. Were all (non-noisy) channels used? Or only the 204 gradiometers?

What were the decoding methods used? Are there references for those methods?

The logic of the lines below is opaque:

Lines 110-113: "First, we attempted to decode the scanning day and scanning sequence order from the MEG brain responses (see Figure 1b). If decoding accuracy was above chance, it would suggest some form of long-term variability in the responses."

Perhaps this is because there is no description of what the baseline is. What is the actual hypothesis one is testing for?

2) Using a single participant

The study is limited to a single person. Could any additional public data, like that in the THINGS-data public database, be used to reinforce the findings of this work? THINGS-data, a multimodal collection of large-scale datasets for investigating object representations in human brain and behavior. Elife, 12, e82580) contains longitudinal MEG data for four participants collected over a few months. Could the authors run similar decoding analyses for animacy within this dataset and compare within- versus across-session decoding there?

3) Variability in visual processing over longer time scales

The curves in Figure 2 show positive and negative values, so they cannot be "explained variance" they look like some representation of best channel average signal (black line) and the corresponding signal from the bootstrap (thin green lines), with clear onset (~ 100 ms) and offset (after 600 ms).

4) Decoding

What is meant by "decode the animacy"? Is decoding the animacy testing whether the MEG signal of a given trial one could determine whether the stimulus presented was animated or not? If this is done at a particular latency, what data were used? All the MEG data of that latency across all the channels? Was it a range latencies used (around the center latency). Could the decoding be performed from the magnetometers only, gradiometers only? Was their improvement if the same number of channels were used but taken in equal numbers from the magnetometers and gradiometers?

Could the authors look at other decoding measures? e.g. individual stimulus level decoding. Does that also appear to be as stable? We do expect some effects of repetition on measured responses.

5) Figure 3

The lines for the decoding in Figure 3 are much smoother for cross scanning day than for within scanning day. Is this because there are many more data points for cross scanning day. This should be made clearer.

Are the axis labels for Figure 3C correct? The horizontal axis does not appear to be days between scans. Perhaps it is the latency so what is shown is the Pearson's r as a function of latencies, not as a function of days between scans.

6) Discussion of Roth and Merriam (2023)

Roth and Merriam (2023) are cited for representational drift of fMRI responses in human V1, but the authors state:

Lines 61-63: "However, it remains unclear whether such changes affect higher-level representations, such as object categories, or whether these representations remain stable despite lower-level fluctuations."

Note that Roth and Merriam (2023) found that in spite of drift in the fMRI responses, representational similarity remained stable:

"We found that despite ongoing changes in single image representations, correlations between RDMs did not diminish over time (Fig. 3G-I), indicating that the RDM remained stable across sessions. This suggests that, while the representation itself is dynamic, the position of representations in stimulus space (i.e., relative to other stimuli) remains constant. Such stimulus-space stability could enable downstream cortical areas to read out an accurate perceptual representation in the face of drifting patterns of activity within V1."

Minor Issues:

Lines 10-12: "The participant passively attended to visual stimuli and with exactly equal stimulus input within each session." What is meant by "exactly equal stimulus input"? Was it meant to be "Similar type stimuli of exactly equal duration" .. or something similar?

## Author Response

We want to thank the reviewers for the constructive review. In what follows, we address each of the reviewers' comments.

Stimuli and procedures This subsection does not provide enough detail for what was done and when. There are many other ways to provide the information that the reader needs to have to appreciate what was done and how. The manuscript should more details and specific examples of the stimuli presented. We suggest the inclusion of a figure and a table. The figure should show the sequence of events and details during one recording day (preparation, time of resting state data before and after (if such data were recorded), and the chronology of the 8 days of measurement (including the one with insufficient trials). The Table should provide the duration of the scan, duration, number of presentations of each type and all details (is it 118 once so about 2 minutes in total? Different each day of recording, any ISI between images) any baseline recordings (e.g. resting state, eyes open/closed?).

To address these comments, we have made the following changes: • Added an example train of stimuli to Figure 1a, replacing the previous icons used to illustrate that both animate and inanimate stimuli were presented. • Added a table (see Table 1) detailing information about each of the scanning days (scanning day, days after first scanning day, presentations per stimulus, total recording duration; no resting-state was collected). • We refer to the text where information about the ISI and proportion of animate and inanimate images can be found: "The participant was instructed to merely attend to the stimuli presented, consisting of 118 images [22] with 27 images of animals (e.g., dog, bird, frog, polar bear) and 91 inanimate objects (e.g., washing machine, strawberry, car, table). Each image was presented for 500 ms, followed by a 400- 500 ms fixation cross (see Figure 1a)." • Revised the description of the session structure and stimulus presentation in the Stimuli and procedure section: "During each session within a scanning day, the participant viewed 118 different real-world images (see examples in Figure 1a). Each image was presented 4 times each session lasting ~13 min. Multiple sessions were completed each scanning day with a small break between. This resulted in more than 10,000 image presentations (see Figure 1a and Table 1)." • Clarified the procedure for each scanning day in the "Acquisition and preprocessing of MEG data" section: "The position of the MEG chair within the MSR and the height was marked such that it could be placed in the same position before each session. Between sessions within a scanning day, the participant exited the MEG dewar and headcast during short breaks and was repositioned in the headcast before the next session." Acquisition and preprocessing of MEG data More details are needed for the use of headcast:

Lines 94-97: "The data was acquired using a headcast 3D printed to fit the shape of the participant's head from a rendering of structural MRI data [23]. This ensured similar positioning of the head in the scanner across scanning days and minimal movement during data collection." The need to keep the head fixed is obvious for types of analysis which assume that the head is in precisely the same location on each day of recording. This will be the case source space analysis where source localization depends on knowing where the sensors are relative to the brain; it is also necessary if the identity of each sensor is preserved in the analysis across days, which I believe is the case in this work. To go further one should ask: what are the main reasons for using the headcast: s it to fix the location so it is known relative to the sensors? If yes, why not place localization coils on the cast - that would produce an excellent coregistration result. Which can also be used for co-registering to the brain.

Is it to restrict movement? If yes, this will only be true in the two horizontal conditions. However, if the vertical (sitting) position was used it will depend critically on how long the measurements lasted. It is also relevant if supine position was used, but not as critical.

Overall, we used a headcast to maximise the signal-to-noise ratio (both preserving sensor identity across days and reducing movement), since the headcast was available to us. To support the benefit of using a headcast, we refer to Figure 2 B) from the THINGS-MEG dataset [*], which shows low within- and between-scanning day differences in head position with participants seated and with custom-made headcasts.

We note that we did not perform source-space analyses in this study to keep the pipeline as simple as possible, and because source-reconstruction may affect decoding accuracy (reducing it), depending on how we fine-tune the algorithm. [*] Hebart, M. N. et al. THINGS-data, a multimodal collection of large-scale datasets for investigating object representations in human brain and behavior. eLife 12 (eds Barense, M., de Lange, F. P., Konkle, T. &Glerean, E.) Publisher: eLife Sciences Publications, Ltd, e82580. issn: 2050-084X. https://doi.org/10.7554/eLife.82580 (2025) (Feb. 2023) The next line is a tautology, unless one of two explanations Lines 99-100: "Signal space projections were projected out. ..." a) Some projection was made. In which case the sentence should be "Signal space projections was used to project out .... ." With the "..." stating what noise or other components were removed. b) Signal space projection was used to transform different head locations to one for use across all runs/days, in which case more details should be explained.

We thank the reviewer for pointing out that the original phrasing was unclear. In our preprocessing pipeline, signal space projection (SSP) was applied using projectors estimated from empty-room recordings to remove environmental noise components. The manuscript has been revised accordingly and now reads: "Signal space projection was applied using projectors estimated from empty-room recordings to remove environmental noise components." We did not use SSP for head position transformation across runs or days.

Lines 100-101: "Bad channels were identified by visual inspection and interpolated to ensure an equal number of features across scanning days." Please provide the range of how many bad channels were encountered across the different days of recordings.

We have now added this information to the Acquisition and preprocessing of MEG data section: "Bad channels were identified by visual inspection and interpolated to ensure an equal number of features across sessions (between 1 and 11 removed, µ= 6.06)". As each scanning day has more sessions, we specify the information per session, rather than per scanning day.

Lines 102-106: "To better facilitate identification of artefacts [25], a stronger high-pass filter at 1 Hz was applied to the data. ... Additionally, the data was low-pass filtered at 40 Hz. ..." What is meant by a "stronger high-pass filter at 1 Hz"? Reference 25 does not provide any information because it cannot be found (the title is missing from the definition provided). The filtering confines the search to relatively low frequencies, so the results demonstrated are relevant to that range (1-40 Hz) band, some more information about the kind of filters used would help check whether the filtering has other consequences, for example if by strong filtering it is meant sharp cut-off.

Thank you for pointing this out. The wording "stronger high-pass filter" was imprecise and has been removed. The Acquisition and preprocessing of MEG data section has now been modified and now reads: "The data were high-pass filtered at 1 Hz to reduce slow drifts and low-pass filtered at 40 Hz using linear-phase FIR filters implemented in MNE-Python." Consequently, the reported results reflect neural activity within the 1-40 Hz range.

Reaching the end of subsection 2.2, it is confusing for what pre-processing operations (filtering, eye and heart artifact eliminations) were visual inspection, signal space projection and PCA used. Could this be all put in one place and clearly defined what method was used for what pre-processing step? We have now rewritten this section to make it clearer: "The preprocessing of MEG data was carried out in MNE-python [24] and consisted of the following ordered steps. Signal space projection was applied using projectors estimated from empty-room recordings to remove environmental noise components. Bad channels were identified by visual inspection and interpolated to ensure an equal number of features across sessions (between 1 and 11 removed, µ= 6.06). The data were high-pass filtered at 1 Hz to reduce slow drifts and low- pass filtered at 40 Hz using linear-phase FIR filters implemented in MNE-Python. Independent component analysis (ICA) was conducted to identify and remove artefacts. Cardiac and ocular components were manually identified and removed, based on inspection of component topographies and time courses (1-4 components per session; µ=2.36). The data were then segmented into epochs from stimulus onset to 1 s post- stimulus and resampled to 250 Hz.

Analysis There is important information missing in this section.

For the one equation provided there is no statement about which data were used.

We assume the Reviewer refers to the Equation defining explained variance. We have now clarified what y exactly represents: "Where y represents the ground-truth scanning day or scanning order, y_pred is the out-of- sample predicted scanning day or scanning order and y_bar is the mean of y." In section 2.2 it is stated that the recordings were made with the NeuroMaGOy system with 305 channels. Were all (non-noisy) channels used? Or only the 204 gradiometers? Both magnetometers and gradiometers were used for decoding, expecting that the prediction algorithm would be able to filter the relevant information in a data-driven way. We have now stated this clearly for both decoding analyses: • "Decoding was performed separately at each time point from stimulus onset to 1000 ms post-stimulus. At a given time point, the feature vector for each trial consisted of the MEG signal across all gradiometers and magnetometers." • "At a given latency, each trial was represented by the MEG signal across all sensors at that time point." What were the decoding methods used? Are there references for those methods? We thank the reviewers for pointing out that this crucial information was missing for the cross-scanning day decoding. We have now added this information: "Decoding analysis was performed using linear discriminant analysis (LDA) [28] using scikit-learn [26] and repeated for each time point within the trial. At a given latency, each trial was represented by the MEG signal across all sensors at that time point.". Additionally relevant references for the decoding scanning day and scanning order analyses has been added: "To evaluate whether the processing of identical stimuli varies over long time scales, we used linear ridge regression [25] as implemented in scikit-learn [26] to predict two variables from the MEG data: the calendar day of the scan and its position within the sequence of scanning days (scanning order)." The logic of the lines below is opaque:

Lines 110-113: "First, we attempted to decode the scanning day and scanning sequence order from the MEG brain responses (see Figure 1b). If decoding accuracy was above chance, it would suggest some form of long-term variability in the responses." Perhaps this is because there is no description of what the baseline is. What is the actual hypothesis one is testing for? We have completed the text to clarify the logic of this as follows: "First, we attempted to decode the scanning day and scanning sequence order from the MEG brain responses (see Figure 1b). The idea is that if there is a systematic, monotonic change in the signal throughout time (in the order of weeks or months), then the signal should be statistically significant predictor of time itself -either scanning order or day. On the contrary, if there is not any long-term variability in the signal (or if the change is not monotonic), then we should not be able to predict time above chance." Using a single participant The study is limited to a single person. Could any additional public data, like that in the THINGS-data public database, be used to reinforce the findings of this work? THINGS- data, a multimodal collection of large-scale datasets for investigating object representations in human brain and behavior. Elife, 12, e82580) contains longitudinal MEG data for four participants collected over a few months. Could the authors run similar decoding analyses for animacy within this dataset and compare within- versus across- session decoding there? We have now expanded the paper by analysing this dataset. We added a description of THINGS along with some minimal changes to the analyses to accommodate this different dataset to the methods section: "To validate and compare our results, we repeated the analyses on a similar dataset from Hebart et al. [29]. The THINGS-MEG dataset consists of MEG recordings from four participants who viewed images drawn from the THINGS database [30], which comprises 1,854 object concepts represented by 26,107 naturalistic images. The data were recorded using a CTF 275 MEG system (CTF Systems, Canada) with a whole-head array of 275 radial 1st order gradiometer channels.

Each participant was shown a representative subset of these images, distributed across 12 separate recording sessions. Each image was presented for 500 ms followed by a 1 {plus minus} 0.2 s fixation cross. Similar to our dataset, participants wore custom headcasts, and head positions were found to be consistent both within and across scanning days.

The THINGS-MEG dataset differs from our dataset in two key respects. First, in our dataset, the same images were presented multiple times within each scanning day, whereas in THINGS-MEG each image was shown only once. Second our dataset spans a longer total recording period (161 days), whereas THINGS-MEG was collected over shorter intervals of 29, 32, 43, and 72 days for participants 1-4, respectively." Additionally, we have added two supplementary figures (Extended Data Figures 2-1 and 2- 2) which show the participant level result for each of the 4 participants comprised in the THINGS-MEG dataset. As described in the revised manuscript: "We find significant time-points for participants 1, 2, and 4 of the THINGS dataset (see Extended Data Figures 2-1 and 2-2)" To assess whether increasing the time between training and testing sessions affected performance, we also report: "No significant clusters were found, indicating that increased time between training and testing was not associated with poorer performance. No effect was found in either of the four participants in the THINGS-MEG dataset either (see Extended Data Figure 3-1)." We have also integrated the THINGS results into the discussion section: • "Extending these findings, we analysed an additional four participants in the THINGS dataset. Notably, no exact stimulus images were repeated (although pictures of the same object categories were shown). Despite the absence of image repetition both scanning day and scan order could still be predicted from the MEG responses in three of the four participants. This indicates that the temporal structure in the neural data is not driven by stimulus-specific repetition but reflects broader changes that generalise across stimuli." • "Cross-scanning day decoding remained stable over time and did not vary systematically with the temporal distance between scanning days. This stability was also observed in the four THINGS participants." Variability in visual processing over longer time scales The curves in Figure 2 show positive and negative values, so they cannot be "explained variance" they look like some representation of best channel average signal (black line) and the corresponding signal from the bootstrap (thin green lines), with clear onset (~ 100 ms) and offset (after 600 ms).

In fact, the curves show the explained variance computed for each bootstrap iteration in green using the equation shown in section 2.3.1. The black line is then average of all the green bootstrap lines. Negative values in explained variance are indeed possible when the squared error from the model prediction is larger than that of the baseline model (see the equation defining EV, where the second term would be greater than 1 in that case). This occurs basically when the model's overfitting is larger than its predictive power. We have added a note to the figure text to clarify this: "Thin green lines represent the explained variance for each individual bootstrap sample. The black line shows the average across bootstrap samples. The explained variance can take negative values when the model performs worse than the baseline model (which is here the mean)." Decoding What is meant by "decode the animacy"? Is decoding the animacy testing whether the MEG signal of a given trial one could determine whether the stimulus presented was animated or not? If this is done at a particular latency, what data were used? All the MEG data of that latency across all the channels? Was it a range latencies used (around the center latency). By "decoding animacy," we mean training a classifier to distinguish between neural responses to animate versus inanimate stimuli. To make this explicit, we have revised the manuscript to read: "Second, we trained models to discriminate between animate and inanimate objects (e.g. a pencil) using data from each scanning day separately." The decoding was done using a single time point from individual trials; that is no range of latencies around the center latency. We have specified this in decoding methods section for both analyses: • "Decoding was performed separately at each time point from stimulus onset to 1000 ms post-stimulus. At a given time point, the feature vector for each trial consisted of the MEG signal across all gradiometers and magnetometers. Thus, each time point yielded an independent regression model, allowing us to track when information about the scanning day and scanning order is present in the evoked response." • "At a given latency, each trial was represented by the MEG signal across all sensors at that time point." Could the decoding be performed from the magnetometers only, gradiometers only? Was their improvement if the same number of channels were used but taken in equal numbers from the magnetometers and gradiometers? In the present analyses, we used data from all sensors as inputs to the models.

Systematically evaluating the relative contribution of magnetometers and gradiometers would be a separate methodological question, which we believe is beyond the scope of the current study. Furthermore, we expect the practical effect of this selection to be relatively minor, because the classifier can to some extent filter out the irrelevant information.

Could the authors look at other decoding measures? e.g. individual stimulus level decoding. Does that also appear to be as stable? We do expect some effects of repetition on measured responses.

We agree that stimulus-level decoding is conceptually interesting. However, the number of trials per individual stimulus for each scanning day was limited (between 12 and 18 presentations per stimulus per day as seen in Table 1). Performing cross-validated, cross- scanning-day decoding at the individual stimulus level would therefore result in very small training sets, even when using leave-one-out cross-validation. For this reason, we focused on the higher-level animate versus inanimate classification, which is also a standard, well- validated contrast in the field.

Figure 3 The lines for the decoding in Figure 3 are much smoother for cross scanning day than for within scanning day. Is this because there are many more data points for cross scanning day. This should be made clearer.

Indeed, there are only 7 scanning days, so we average over fewer accuracy time courses within scanning day (7), as compared to cross-scanning day. We have added the following to the figure text to make this clearer: "Note that the cross-scanning-day average appear smoother as it represents the average over a larger number of pairs compared to within- scanning-days." Are the axis labels for Figure 3C correct? The horizontal axis does not appear to be days between scans. Perhaps it is the latency so what is shown is the Pearson's r as a function of latencies, not as a function of days between scans.

The horizontal axis in Figure 3C represents post-stimulus time (0-1 s), not days between scans. For each time point, we computed the Pearson correlation across all pairs of scanning days between (i) cross-day decoding accuracy and (ii) the temporal distance (in days) between those scanning days. We have now updated the figure text to describe this more clearly: "[C] Time-resolved analysis of long-term stability of animacy decoding. The x-axis shows post-stimulus time (0-1 s). At each time point, we computed the Pearson correlation across all pairs of scanning days between (i) cross-day decoding accuracy at that time point and (ii) the temporal distance (in days) separating the corresponding scanning days. Negative correlations would indicate that decoding performance decreases as the temporal distance between recordings increases. Light green lines show correlations obtained using permuted scanning-day distances (null distribution). No significant clusters (p < 0.001) were identified" Discussion of Roth and Merriam (2023) Roth and Merriam (2023) are cited for representational drift of fMRI responses in human V1, but the authors state:

Lines 61-63: "However, it remains unclear whether such changes affect higher-level representations, such as object categories, or whether these representations remain stable despite lower-level fluctuations." Note that Roth and Merriam (2023) found that in spite of drift in the fMRI responses, representational similarity remained stable: "We found that despite ongoing changes in single image representations, correlations between RDMs did not diminish over time (Fig. 3G-I), indicating that the RDM remained stable across sessions. This suggests that, while the representation itself is dynamic, the position of representations in stimulus space (i.e., relative to other stimuli) remains constant. Such stimulus-space stability could enable downstream cortical areas to read out an accurate perceptual representation in the face of drifting patterns of activity within V1." Indeed, Roth and Merriam (2023) show that correlations between representational dissimilarity matrices (RDMs) remain stable across sessions, indicating preserved relative distances among individual scene stimuli. However, their analysis focuses on stimulus- level relationships among individual natural scene images. In contrast, our question concerns the stability of higher-level representational structure-specifically, object category representations-which involves abstraction across exemplars rather than preservation of pairwise distances among individual stimuli.

To clarify this distinction, we have revised the manuscript to read: "In humans, a recent fMRI study demonstrated representational drift in primary visual cortex (V1) by training encoding models to characterise voxel-level tuning to visual stimuli and testing their generalisation across sessions over the span of a year [20]. The models' predictive performance declined as the interval between training and testing sessions increased, suggesting that the underlying neural representations slowly drifted over time. Notably, however, the representational similarity structure among individual scene stimuli remained stable across sessions, suggesting preserved stimulus-space geometry despite drift in activation patterns.

What remains unclear is whether such voxel-level drift extends beyond stimulus-specific geometry to impact higher-level representational structure, such as object category representations that generalise across exemplars, or whether these more abstract representations remain stable despite lower-level fluctuations. Furthermore, it is unknown how neural responses measured at higher temporal resolution evolve over time scales of weeks or months." Minor Issues:

Lines 10-12: "The participant passively attended to visual stimuli and with exactly equal stimulus input within each session." What is meant by "exactly equal stimulus input"? Was it meant to be "Similar type stimuli of exactly equal duration" .. or something similar? We have now rephrased it to "The participant passively attended to visual stimuli with the same stimulus presented within each session."
