# Supplementary figures and images for: Long-Term Variability in Visual Processing versus Perceptual Stability
Source: eNeuro. 2026 Jun 19;13(6):ENEURO.0344-25.2026. doi: 10.1523/ENEURO.0344-25.2026 (PMC13286707; doi:10.1523/ENEURO.0344-25.2026)

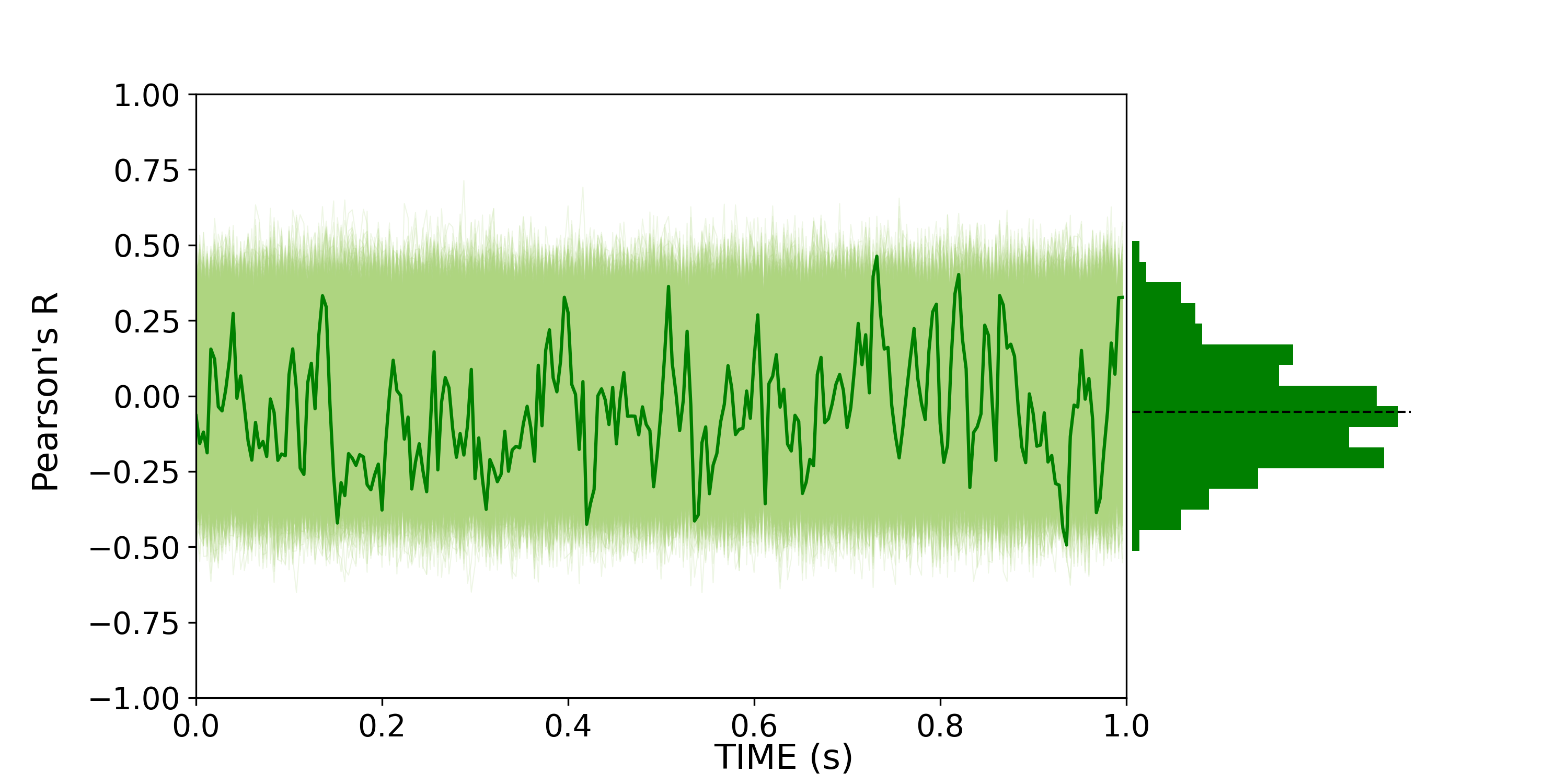

Supplement: Data 1 — Download Data 1, ZIP file. [file eneuro-13-ENEURO.0344-25.2026-s005.zip › VisualVariability-main/decoding/cross_decoding/plots/corr_acc_dist_days.png]

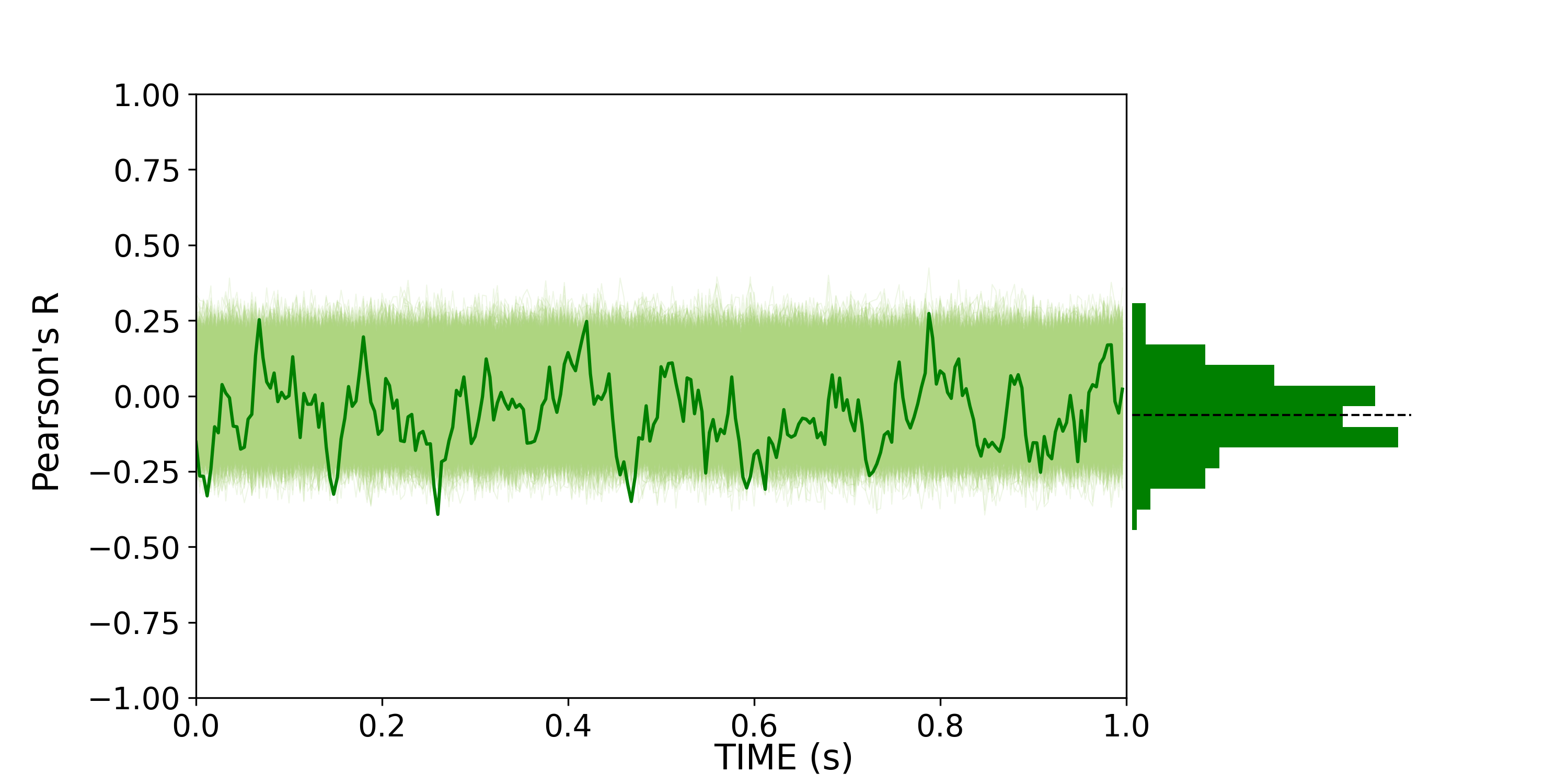

Supplement: Data 1 — Download Data 1, ZIP file. [file eneuro-13-ENEURO.0344-25.2026-s005.zip › VisualVariability-main/decoding/cross_decoding/plots/corr_acc_dist_days_p1.png]

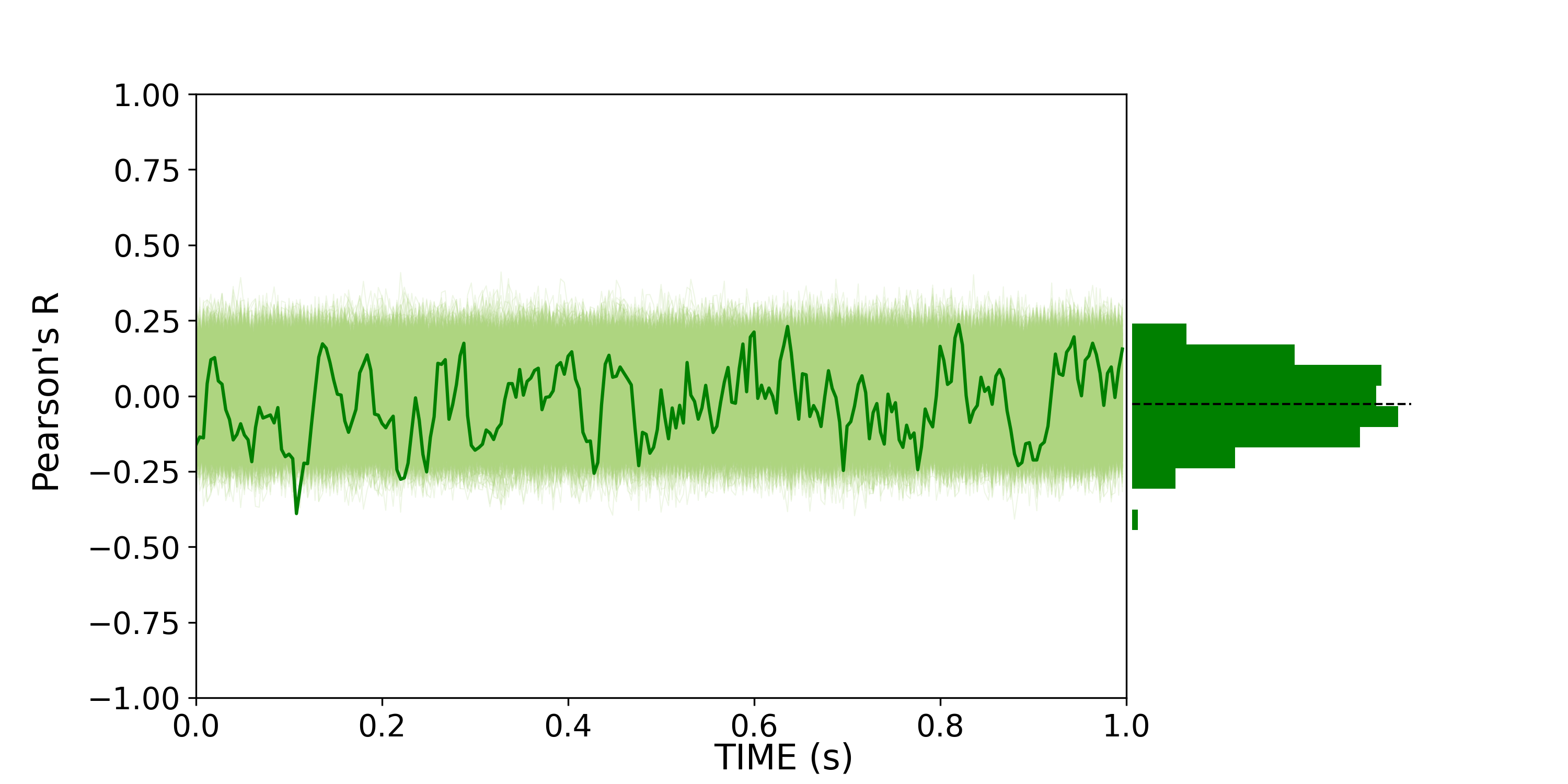

Supplement: Data 1 — Download Data 1, ZIP file. [file eneuro-13-ENEURO.0344-25.2026-s005.zip › VisualVariability-main/decoding/cross_decoding/plots/corr_acc_dist_days_p2.png]

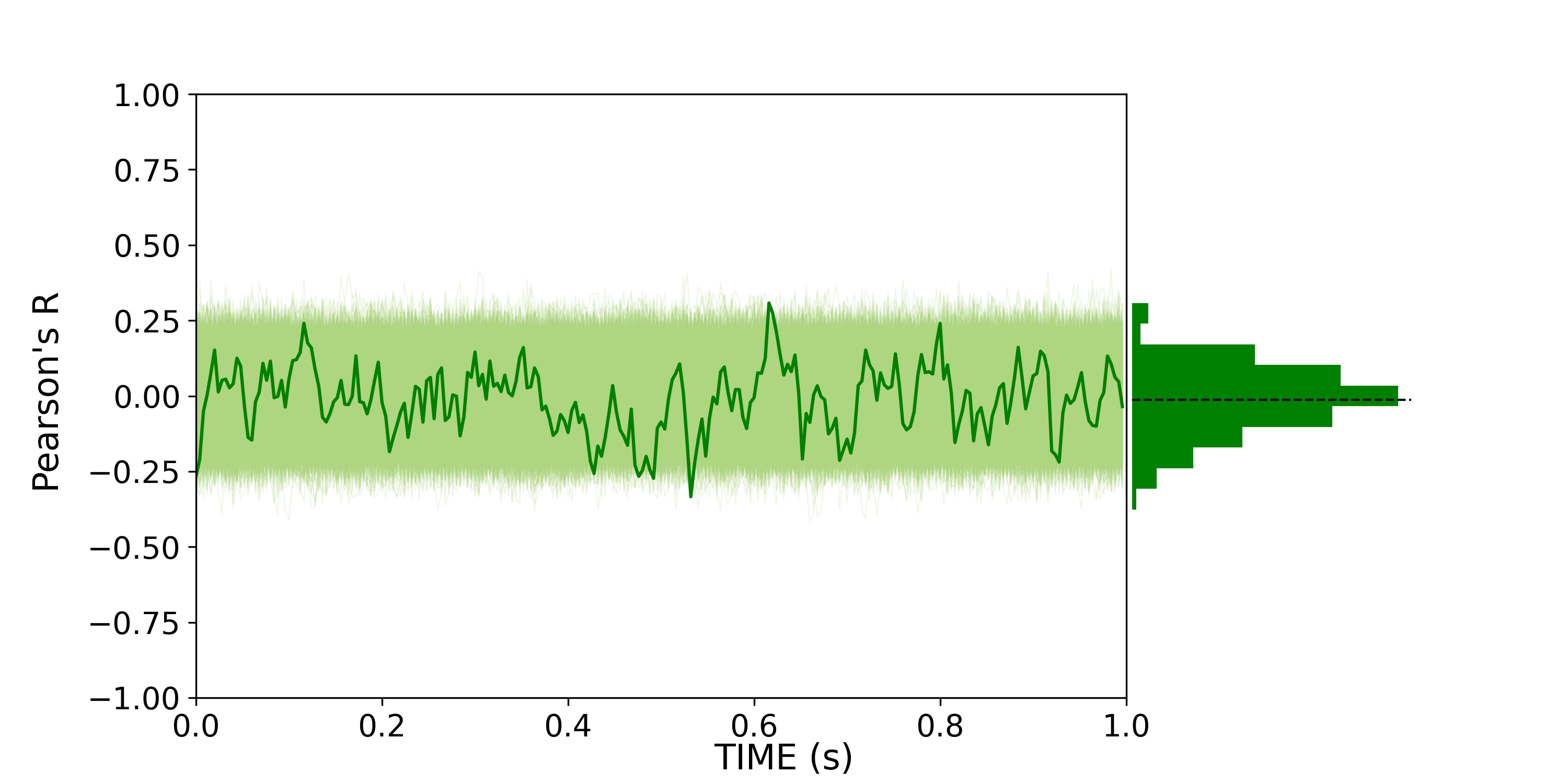

Supplement: Data 1 — Download Data 1, ZIP file. [file eneuro-13-ENEURO.0344-25.2026-s005.zip › VisualVariability-main/decoding/cross_decoding/plots/corr_acc_dist_days_p3.png]

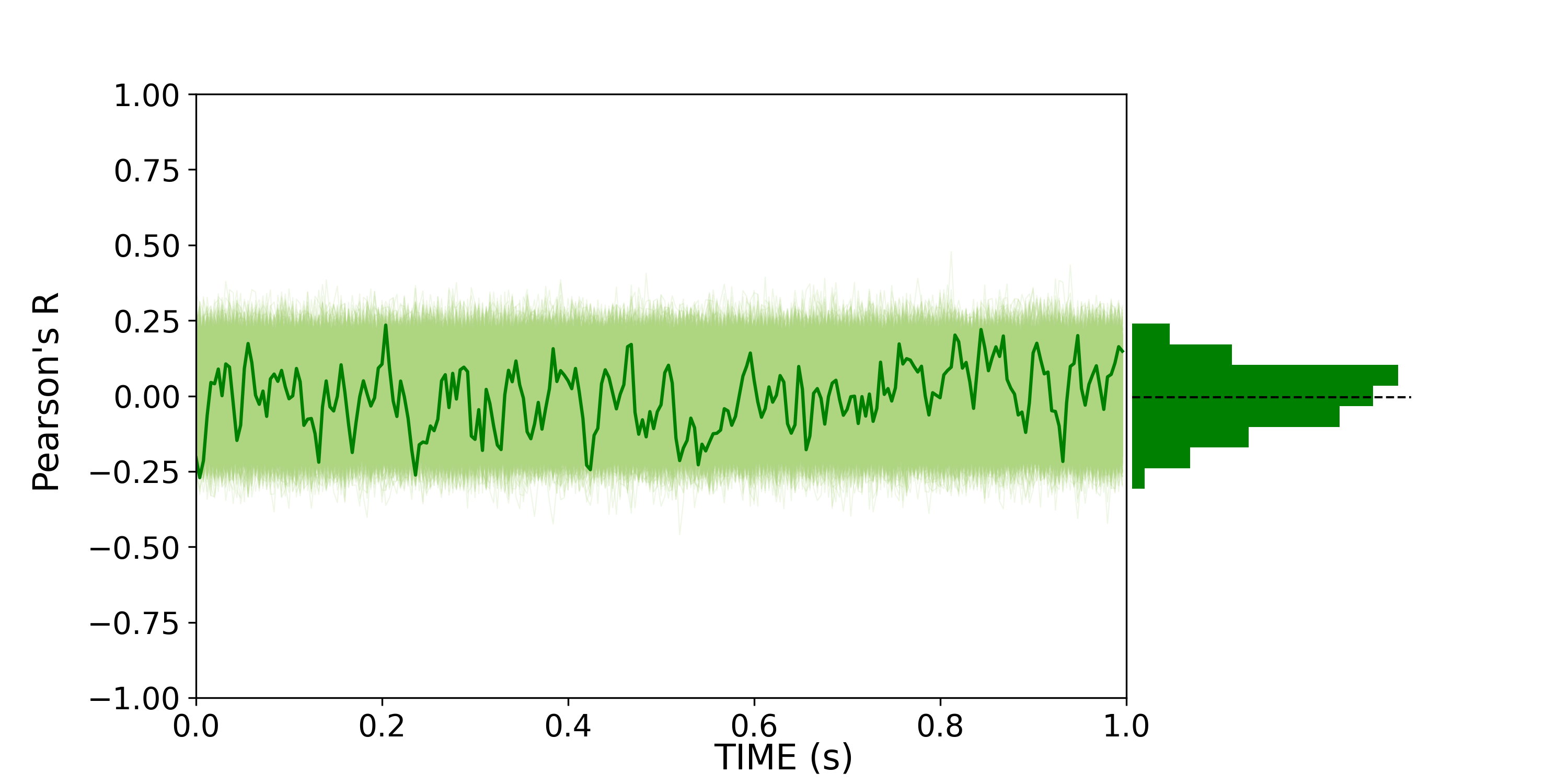

Supplement: Data 1 — Download Data 1, ZIP file. [file eneuro-13-ENEURO.0344-25.2026-s005.zip › VisualVariability-main/decoding/cross_decoding/plots/corr_acc_dist_days_p4.png]

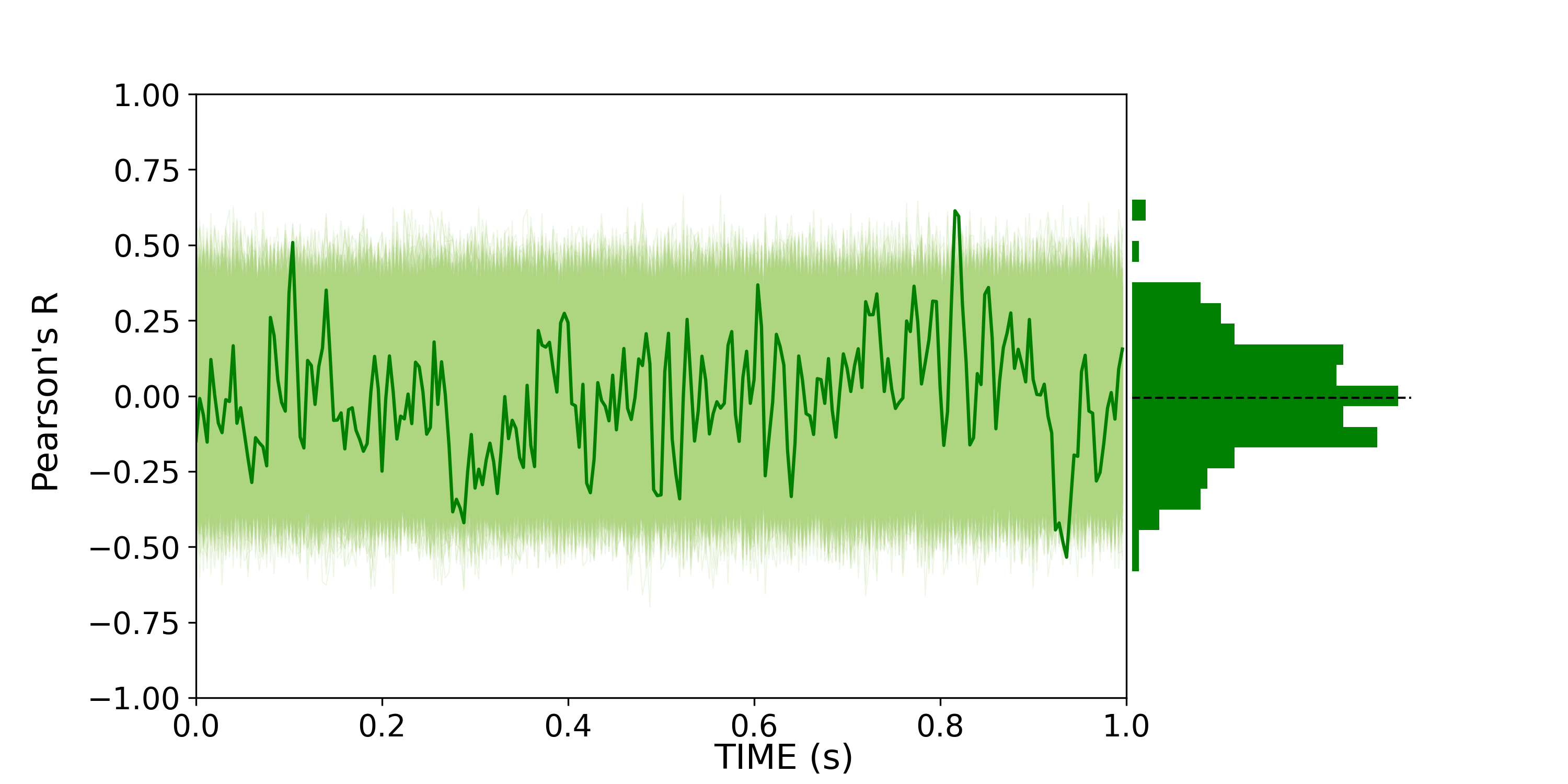

Supplement: Data 1 — Download Data 1, ZIP file. [file eneuro-13-ENEURO.0344-25.2026-s005.zip › VisualVariability-main/decoding/cross_decoding/plots/corr_acc_dist_session.png]

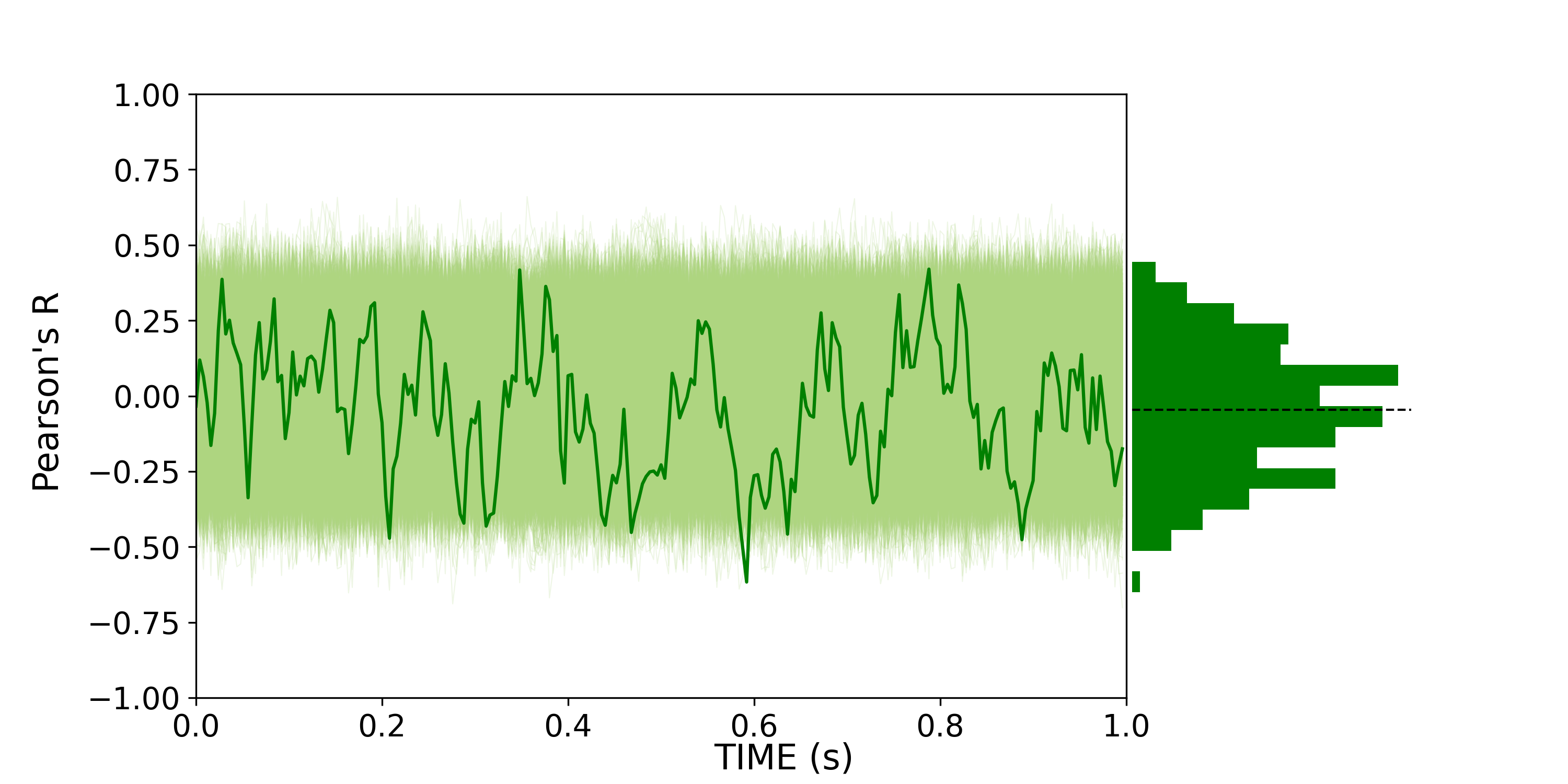

Supplement: Data 1 — Download Data 1, ZIP file. [file eneuro-13-ENEURO.0344-25.2026-s005.zip › VisualVariability-main/decoding/cross_decoding/plots/corr_acc_dist_session_p1.png]

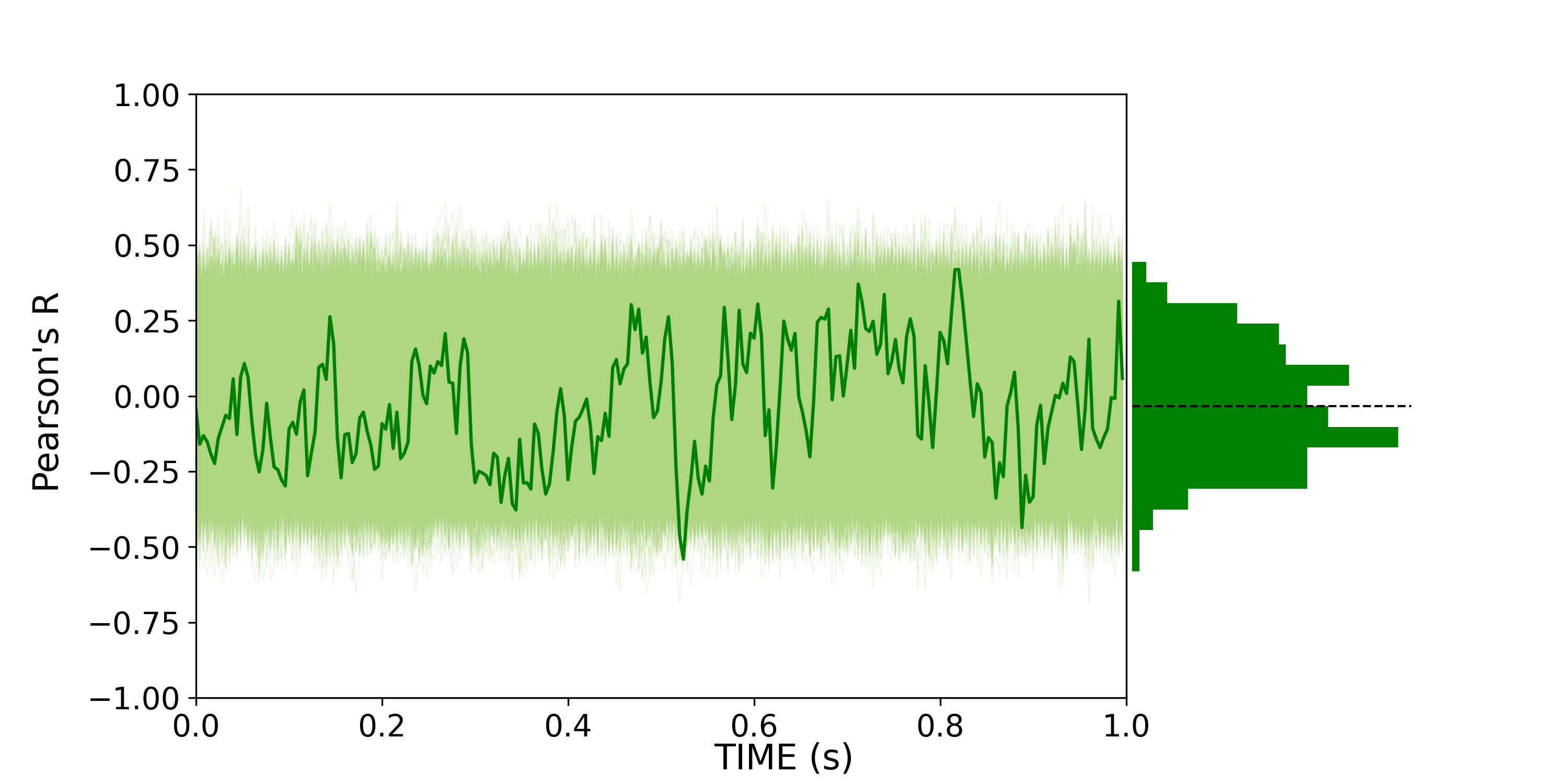

Supplement: Data 1 — Download Data 1, ZIP file. [file eneuro-13-ENEURO.0344-25.2026-s005.zip › VisualVariability-main/decoding/cross_decoding/plots/corr_acc_dist_session_p2.png]

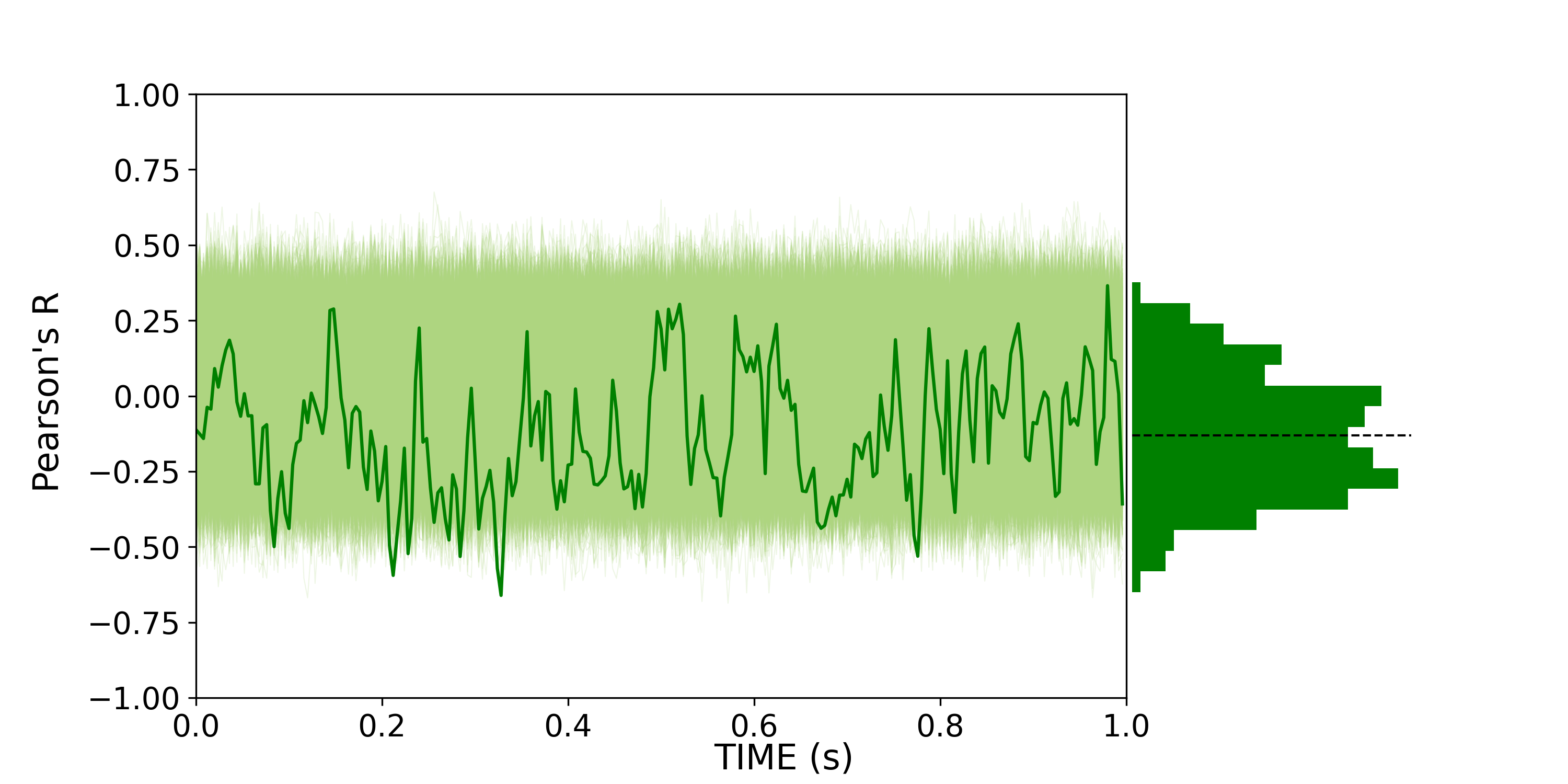

Supplement: Data 1 — Download Data 1, ZIP file. [file eneuro-13-ENEURO.0344-25.2026-s005.zip › VisualVariability-main/decoding/cross_decoding/plots/corr_acc_dist_session_p3.png]

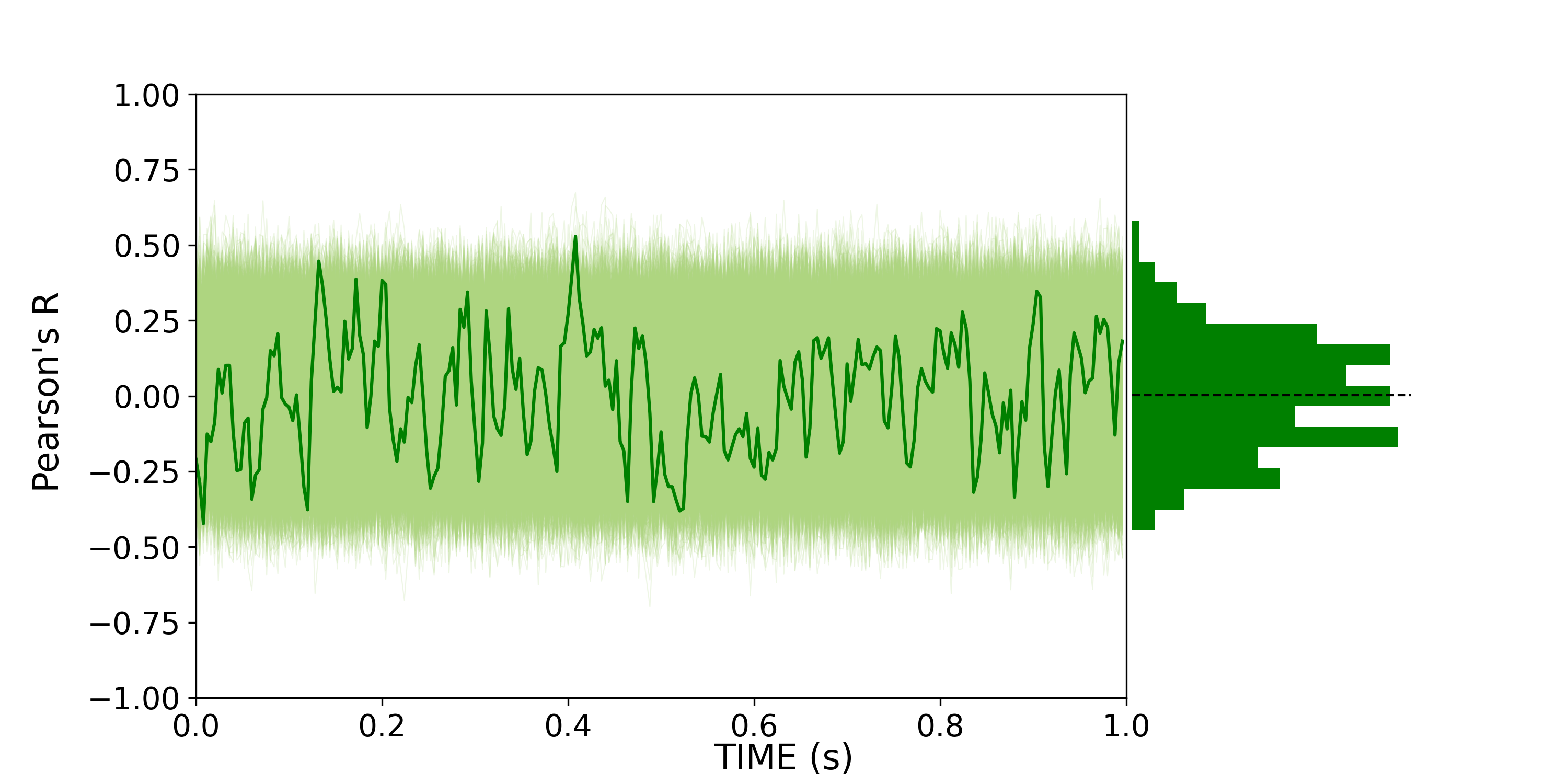

Supplement: Data 1 — Download Data 1, ZIP file. [file eneuro-13-ENEURO.0344-25.2026-s005.zip › VisualVariability-main/decoding/cross_decoding/plots/corr_acc_dist_session_p4.png]

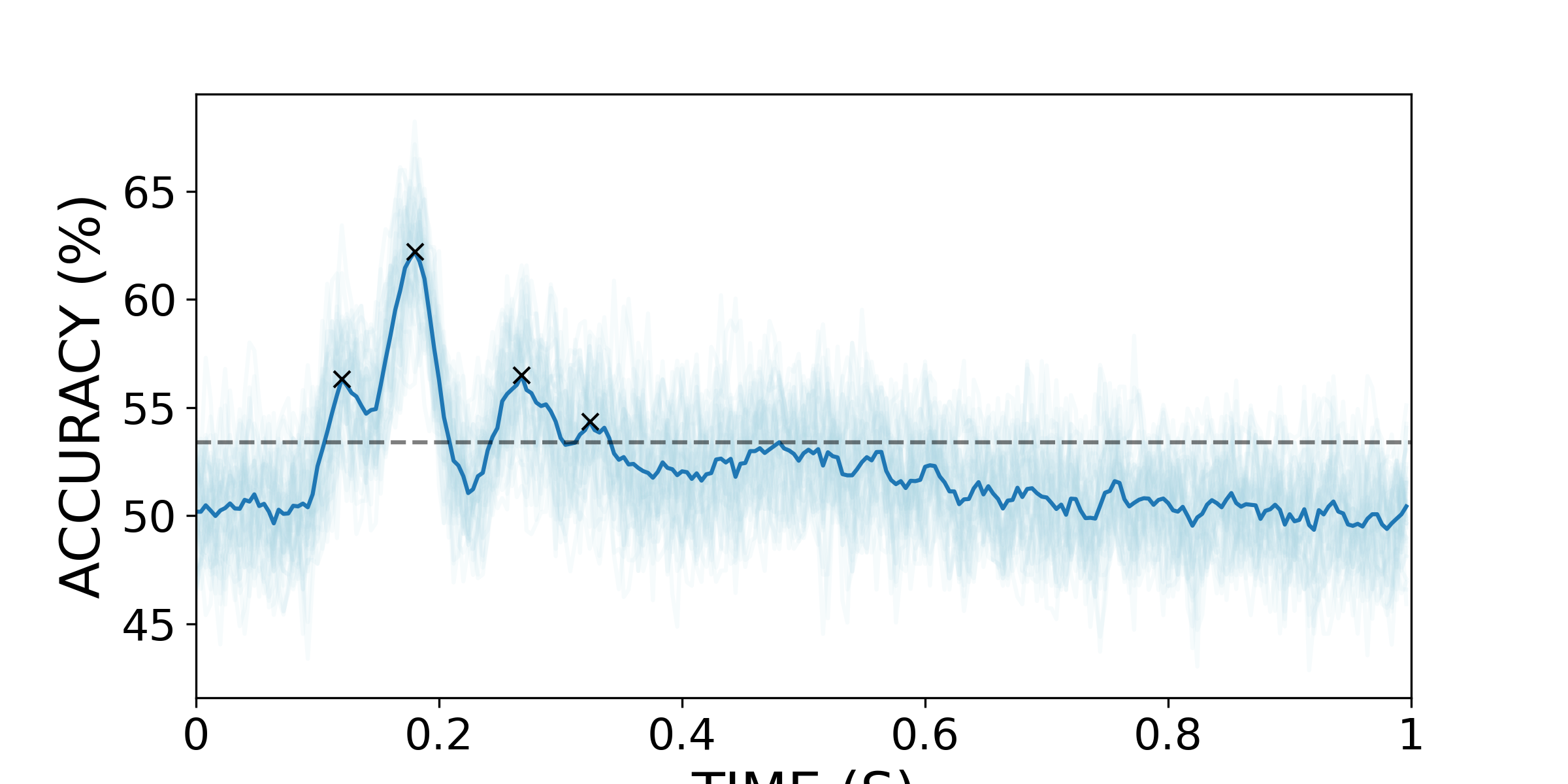

Supplement: Data 1 — Download Data 1, ZIP file. [file eneuro-13-ENEURO.0344-25.2026-s005.zip › VisualVariability-main/decoding/cross_decoding/plots/diagonals_across.png]

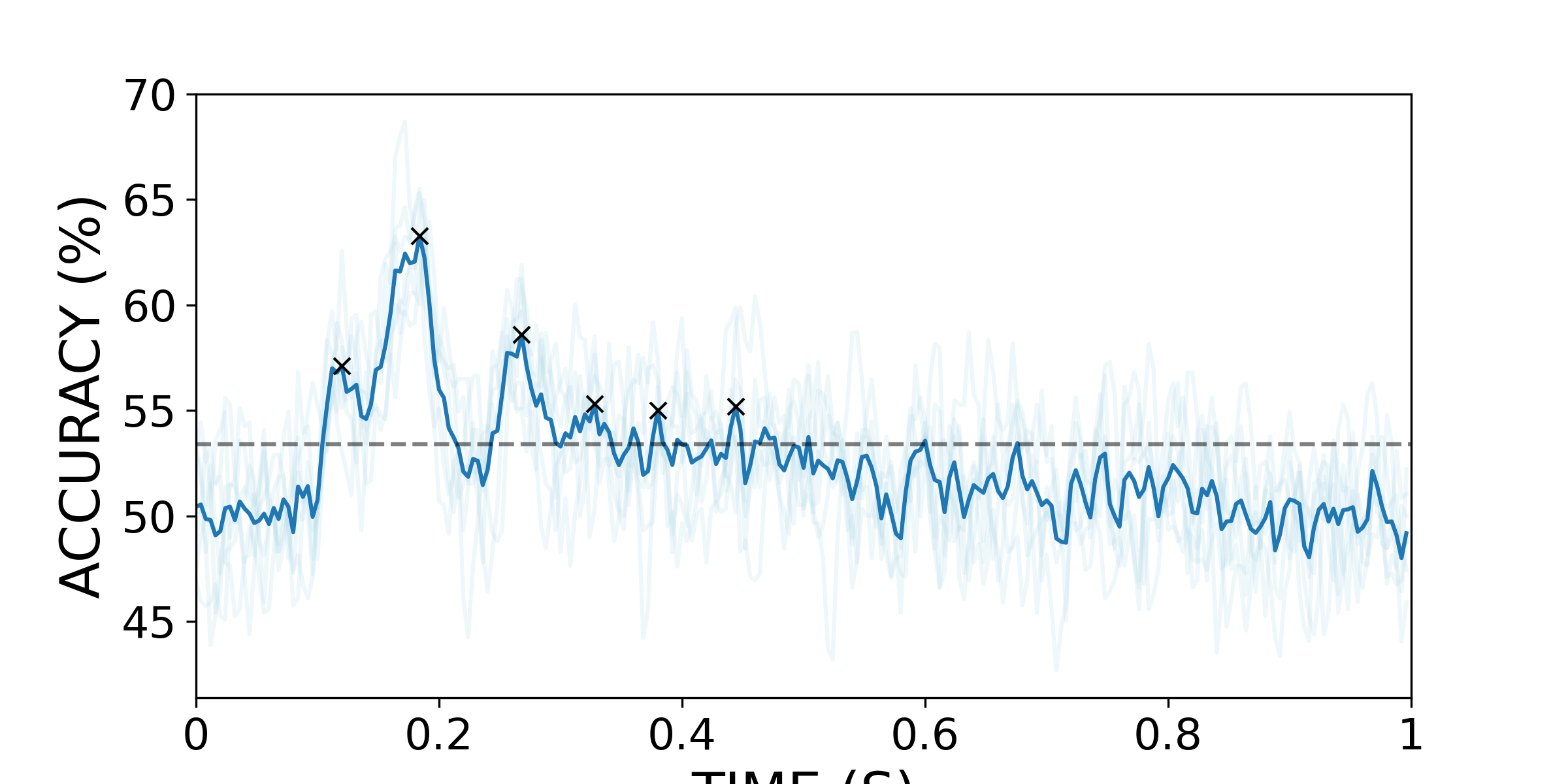

Supplement: Data 1 — Download Data 1, ZIP file. [file eneuro-13-ENEURO.0344-25.2026-s005.zip › VisualVariability-main/decoding/cross_decoding/plots/diagonals_within.png]

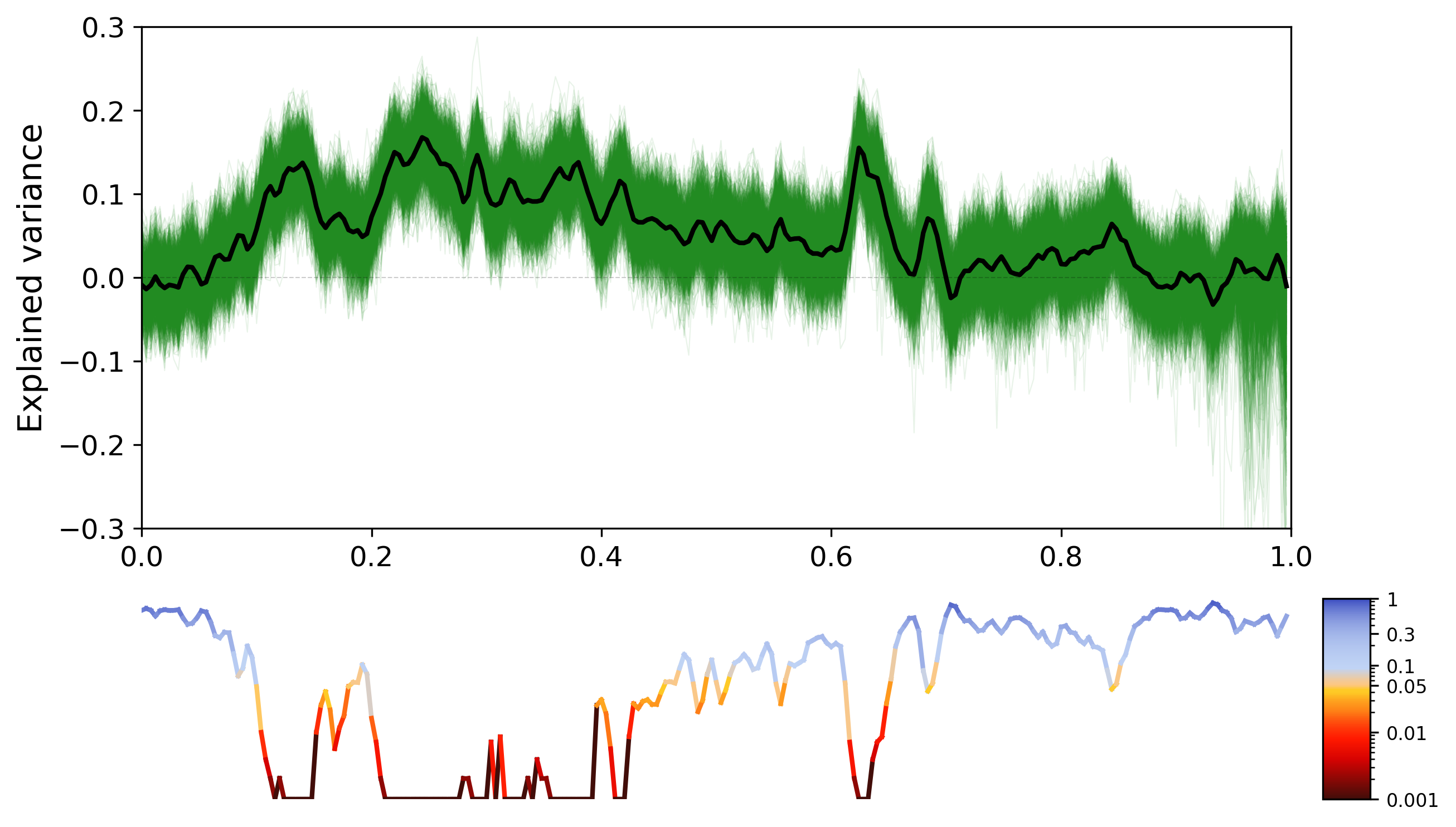

Supplement: Data 1 — Download Data 1, ZIP file. [file eneuro-13-ENEURO.0344-25.2026-s005.zip › VisualVariability-main/decoding/time_elapsed/plots/diagonal_animate_visual_sessionnumber_p2.png]

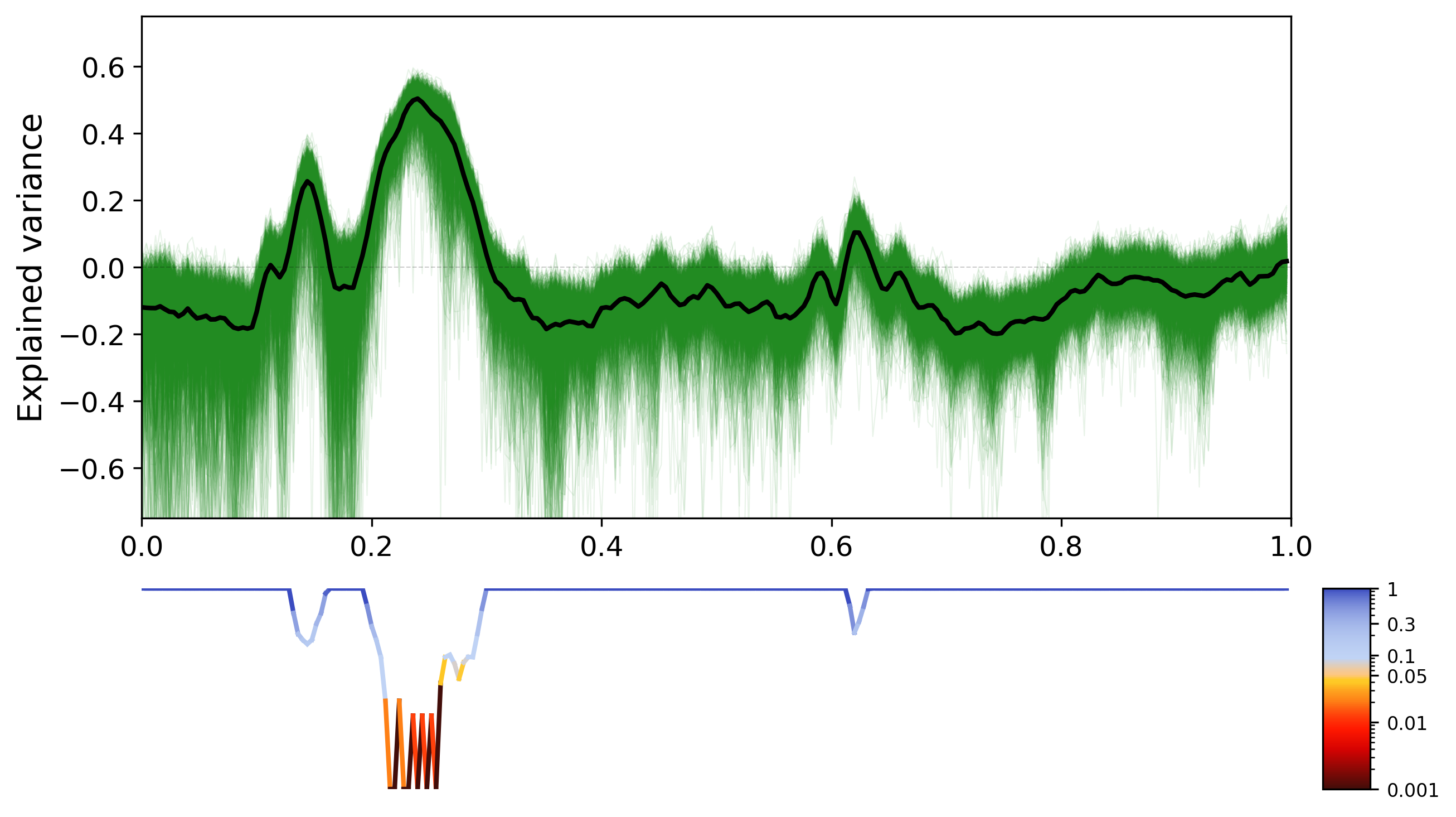

Supplement: Data 1 — Download Data 1, ZIP file. [file eneuro-13-ENEURO.0344-25.2026-s005.zip › VisualVariability-main/decoding/time_elapsed/plots/diagonals_animate_visual_sessionday.png]

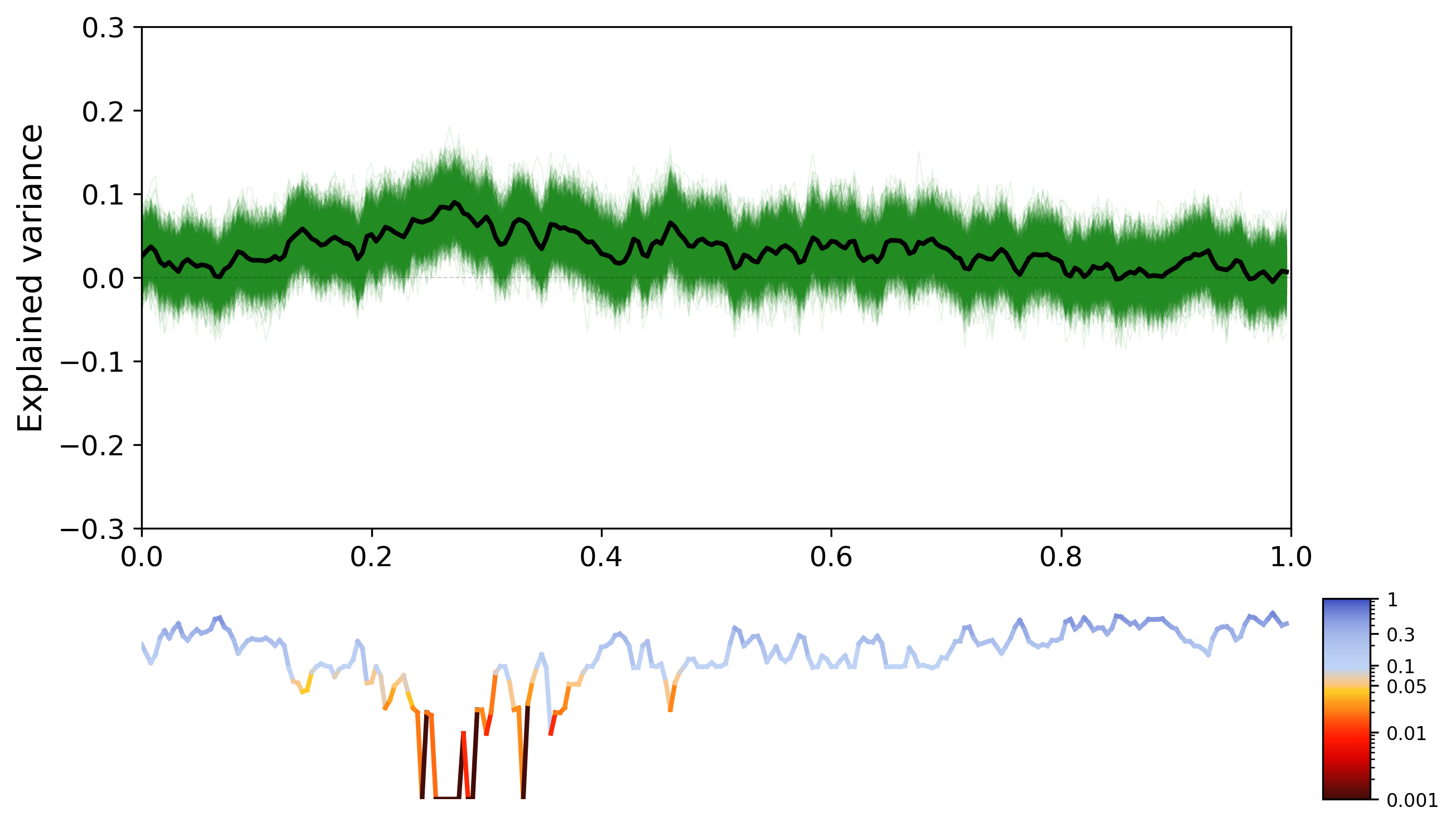

Supplement: Data 1 — Download Data 1, ZIP file. [file eneuro-13-ENEURO.0344-25.2026-s005.zip › VisualVariability-main/decoding/time_elapsed/plots/diagonals_animate_visual_sessionday_p1.png]

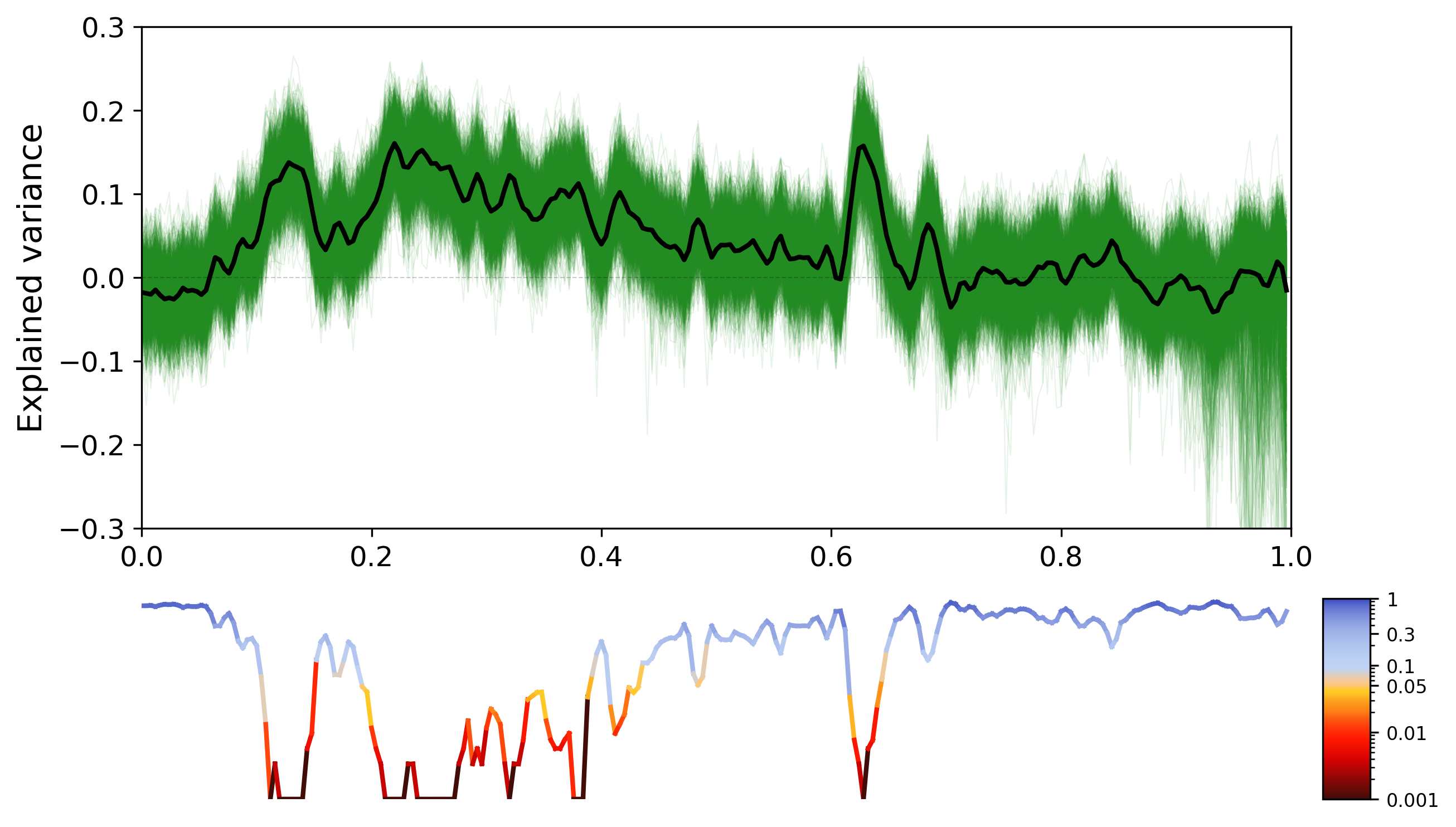

Supplement: Data 1 — Download Data 1, ZIP file. [file eneuro-13-ENEURO.0344-25.2026-s005.zip › VisualVariability-main/decoding/time_elapsed/plots/diagonals_animate_visual_sessionday_p2.png]

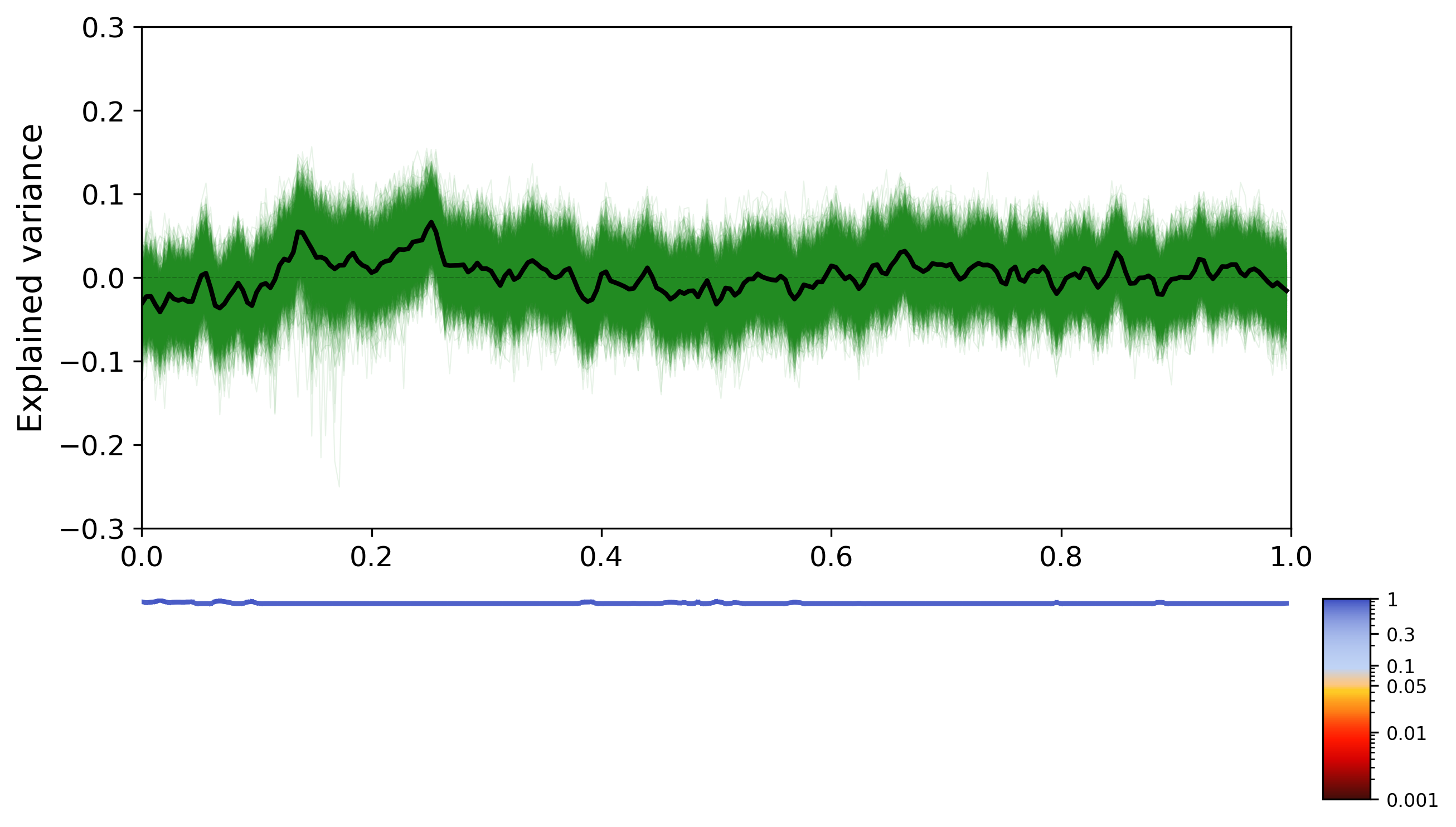

Supplement: Data 1 — Download Data 1, ZIP file. [file eneuro-13-ENEURO.0344-25.2026-s005.zip › VisualVariability-main/decoding/time_elapsed/plots/diagonals_animate_visual_sessionday_p3.png]

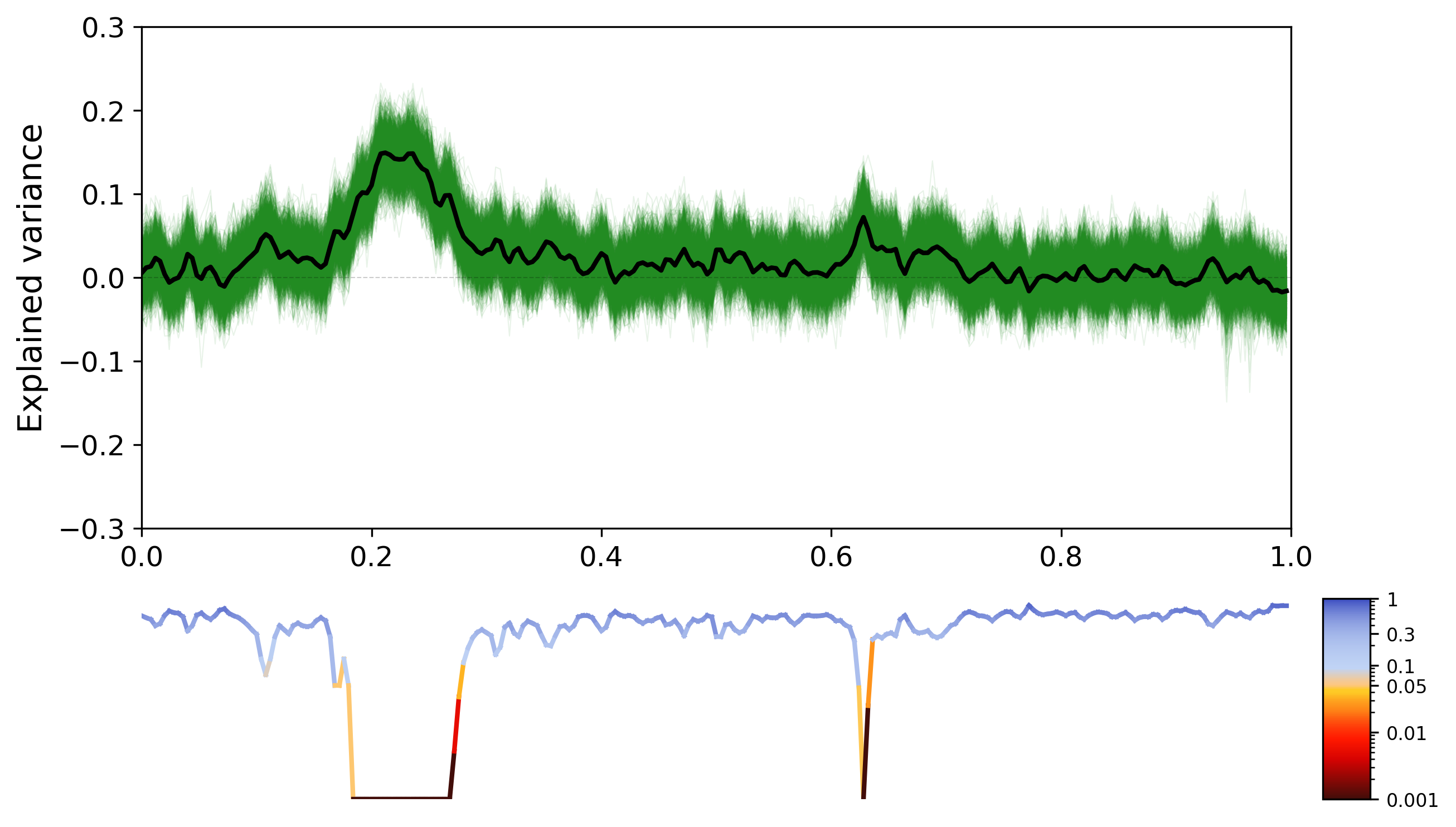

Supplement: Data 1 — Download Data 1, ZIP file. [file eneuro-13-ENEURO.0344-25.2026-s005.zip › VisualVariability-main/decoding/time_elapsed/plots/diagonals_animate_visual_sessionday_p4.png]

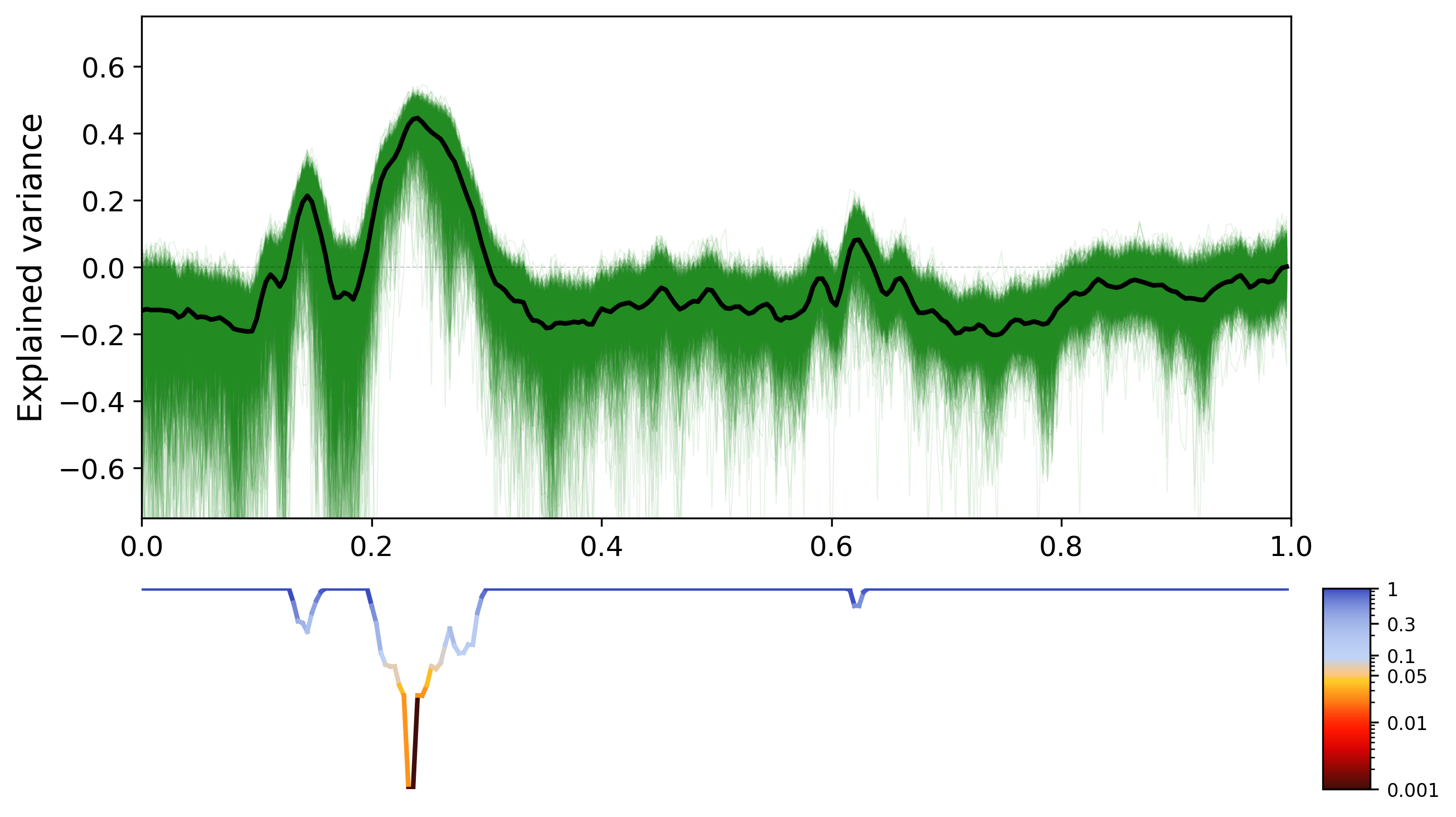

Supplement: Data 1 — Download Data 1, ZIP file. [file eneuro-13-ENEURO.0344-25.2026-s005.zip › VisualVariability-main/decoding/time_elapsed/plots/diagonals_animate_visual_sessionnumber.png]

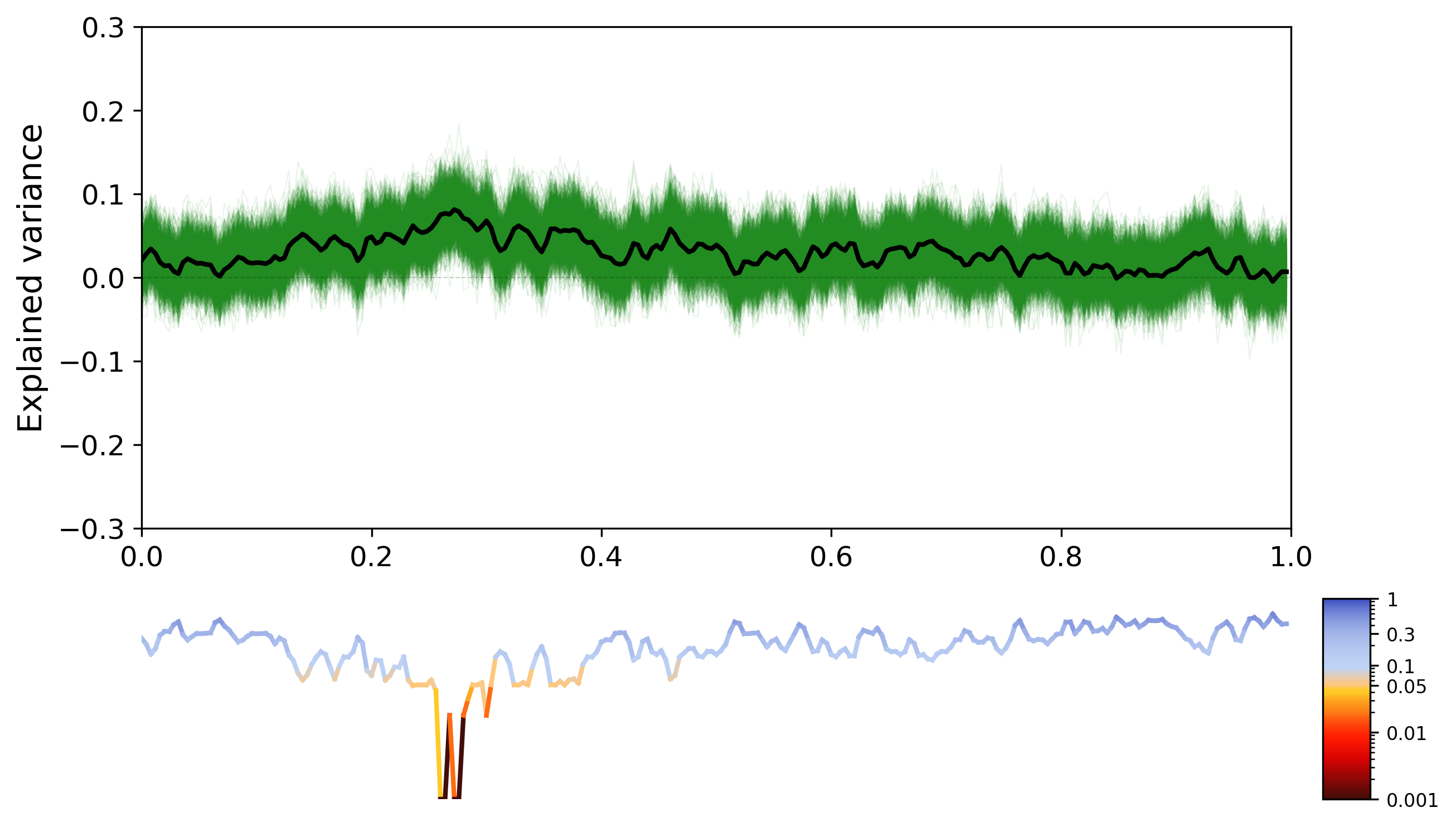

Supplement: Data 1 — Download Data 1, ZIP file. [file eneuro-13-ENEURO.0344-25.2026-s005.zip › VisualVariability-main/decoding/time_elapsed/plots/diagonals_animate_visual_sessionnumber_p1.png]

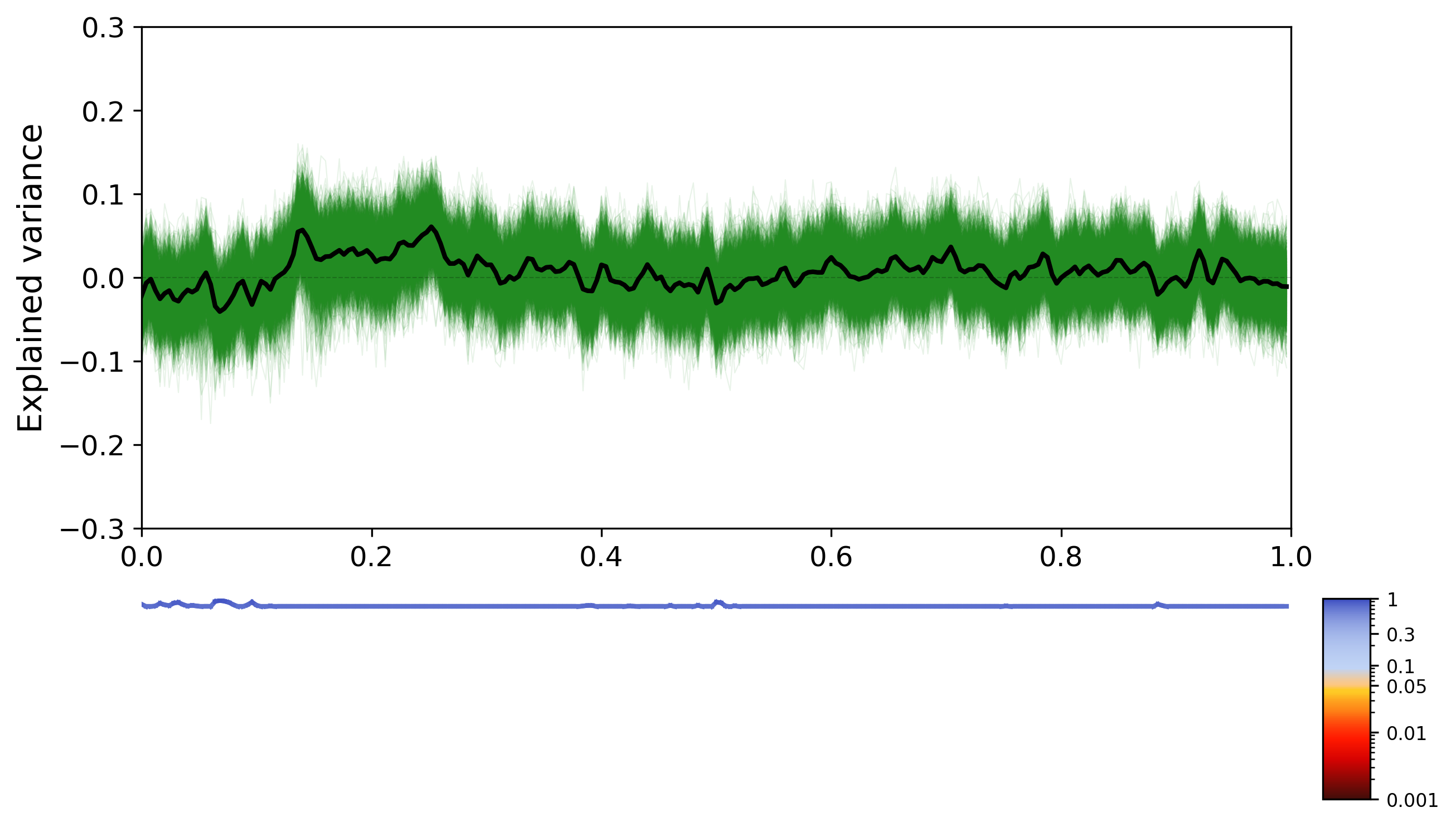

Supplement: Data 1 — Download Data 1, ZIP file. [file eneuro-13-ENEURO.0344-25.2026-s005.zip › VisualVariability-main/decoding/time_elapsed/plots/diagonals_animate_visual_sessionnumber_p3.png]

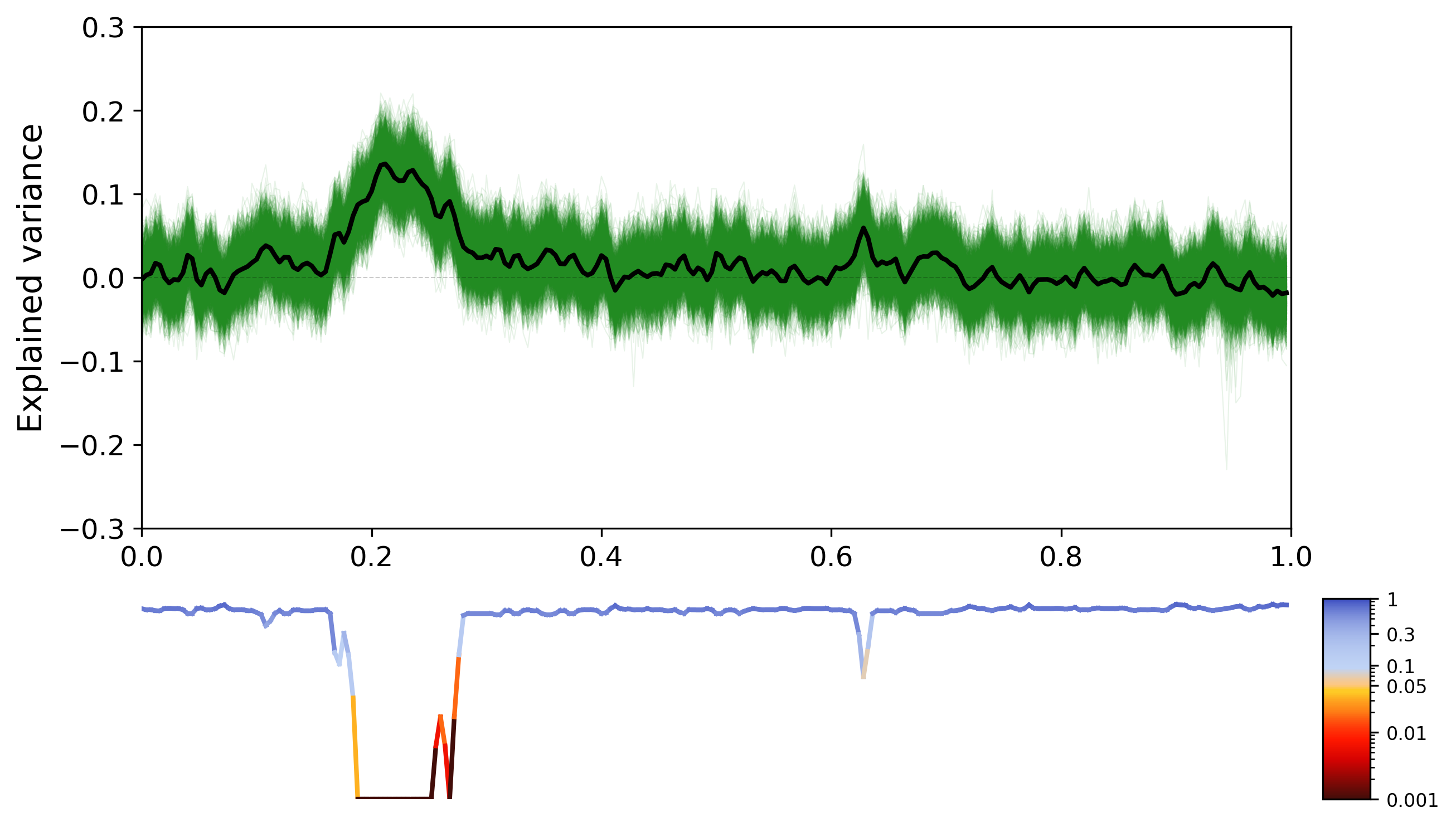

Supplement: Data 1 — Download Data 1, ZIP file. [file eneuro-13-ENEURO.0344-25.2026-s005.zip › VisualVariability-main/decoding/time_elapsed/plots/diagonals_animate_visual_sessionnumber_p4.png]

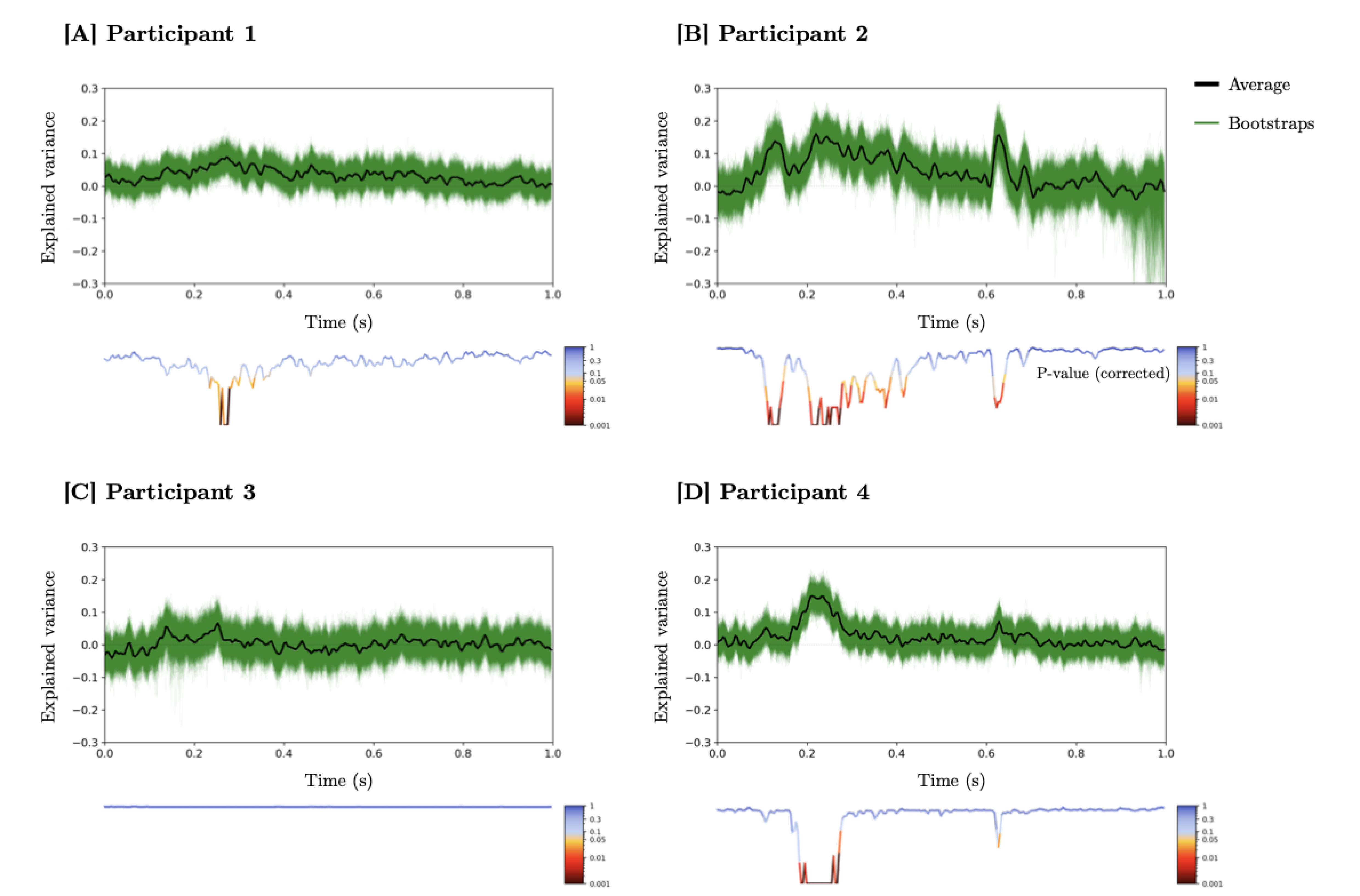

Supplement: Figure 2-1 — Decoding scanning day results for THINGS-MEG dataset. Download Figure 2-1, TIF file. [file eneuro-13-ENEURO.0344-25.2026-s001.tif]

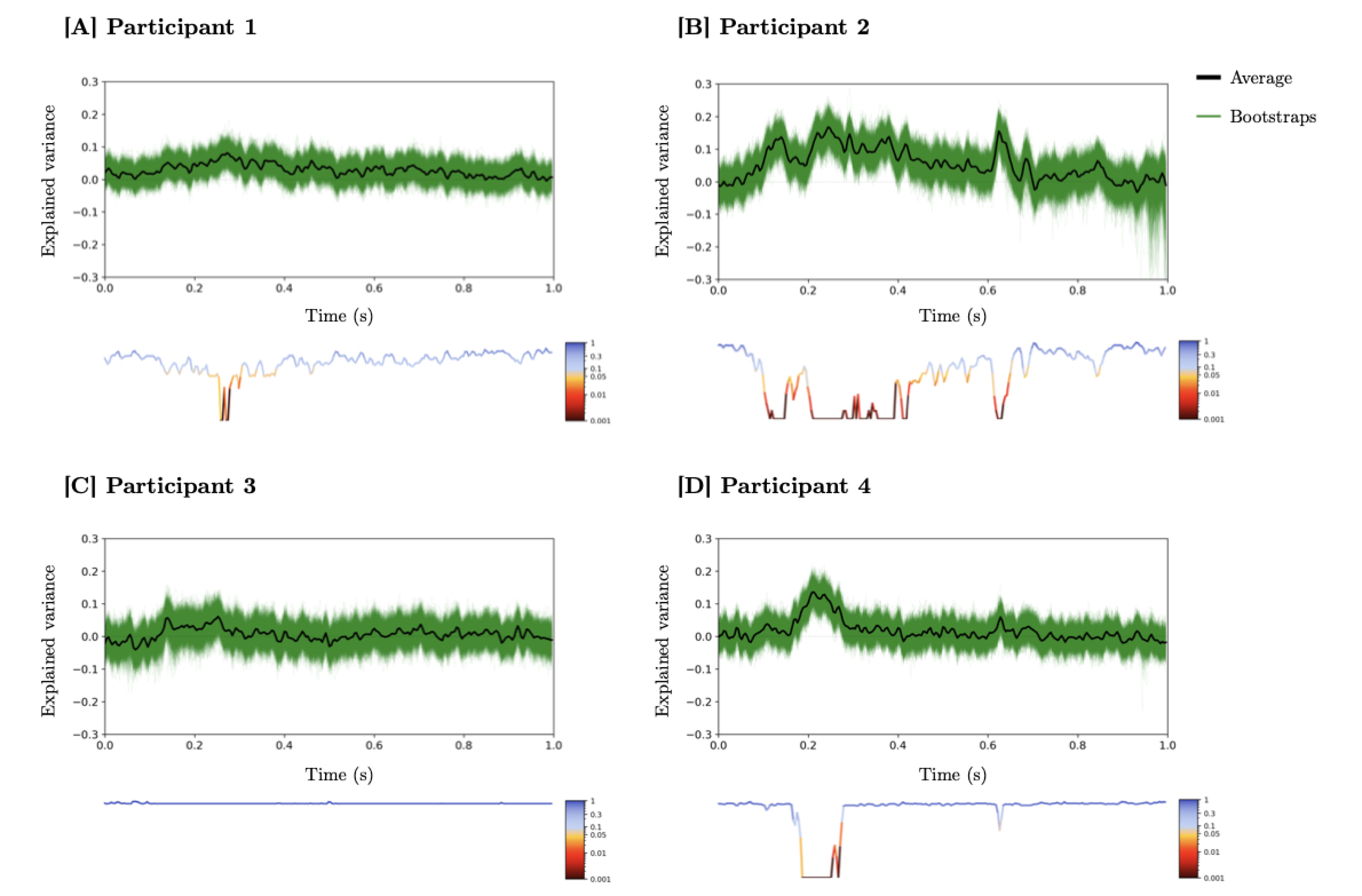

Supplement: Figure 2-2 — Decoding scanning day number results for THINGS-MEG dataset. Download Figure 2-2, TIF file. [file eneuro-13-ENEURO.0344-25.2026-s002.tif]

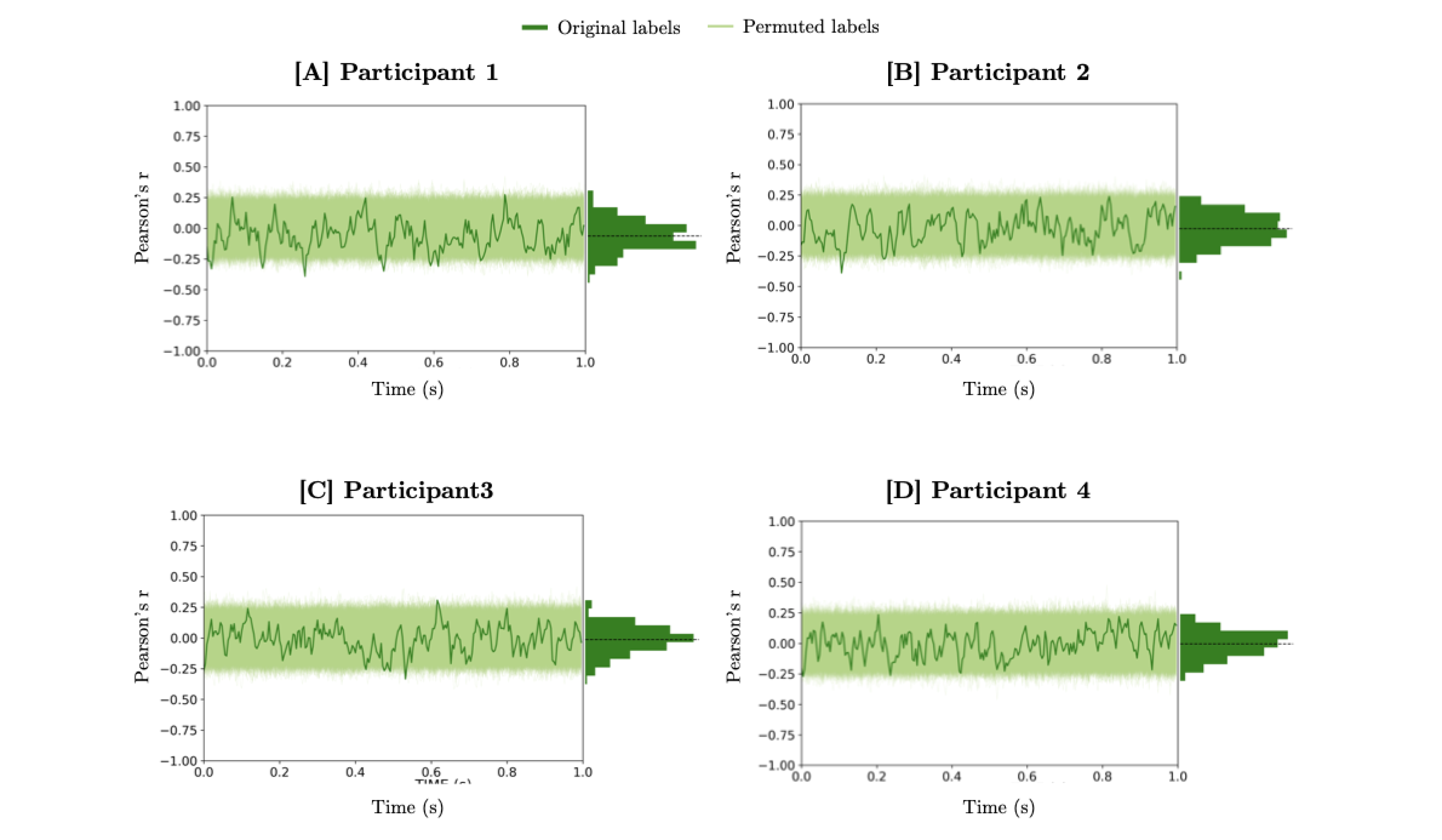

Supplement: Figure 3-1 — Correlation of decoding accuracy and time between scanning days for THINGS data. Same analysis as in Figure 3C. No significant clusters (p < 0.001) were identified for any of the THINGS-MEG participants. Download Figure 3-1, TIF file. [file eneuro-13-ENEURO.0344-25.2026-s003.tif]
